# Synthesis and Antifungal Activity of the Derivatives of Novel Pyrazole Carboxamide and Isoxazolol Pyrazole Carboxylate

**DOI:** 10.3390/molecules20034383

**Published:** 2015-03-09

**Authors:** Jialong Sun, Yuanming Zhou

**Affiliations:** College of Chemistry and Pharmaceutical Sciences, Qingdao Agricultural University, Qingdao 266109, China; E-Mail: sunjialong6289@163.com

**Keywords:** pyrazole carboxamide, isoxazolol pyrazole carboxylate, antifungal activity, synthesis, fungi

## Abstract

A series of pyrazole carboxamide and isoxazolol pyrazole carboxylate derivatives were designed and synthesized in this study. The structures of the compounds were elucidated based on spectral data (infrared, proton nuclear magnetic resonance and mass spectroscopy). Then, all of the compounds were bioassayed *in vitro* against four types of phytopathogenic fungi (*Alternaria porri*, *Marssonina coronaria*, *Cercospora petroselini* and *Rhizoctonia solani*) using the mycelium growth inhibition method. The results showed that some of the synthesized pyrazole carboxamides displayed notable antifungal activity. The isoxazole pyrazole carboxylate **7ai** exhibited significant antifungal activity against *R. solani*, with an EC_50_ value of 0.37 μg/mL. Nonetheless, this value was lower than that of the commercial fungicide, carbendazol.

## 1. Introduction

Phytopathogenic fungi, such as *Rhizoctonia solani* Kuhn, *Alternaria porri* (Ell) Ciferri, *Marssonina coronaria* (Ell.et Davis) Davis and *Cercospora petroselini* Saccardo, pose serious threats to agriculture. They are broad host-range pathogens and infect many crops worldwide, including rice, onions, apples and cucumbers. Many pesticides have been developed and applied to control these diseases with the progression of the modern agrochemical industry. However, the increased microbial resistance of pathogens to known antibiotics facilitates the urgent need for new fungicides [1].

As with many other five-membered heterocyclic compounds, pyrazoles and their derivatives attract increasing attention in the fields of pharmacology and medicine because of their various bioactivities, including antifungal [2], anti-inflammatory [3], antiviral [4], antioxidant [5], cytotoxic [6], antihypertensive [7], A3 adenosine receptor antagonistic [8], antibacterial [9], tranquilizing, psychoanaleptic, muscle-relaxant, hypnotic, antidepressant, ulcerogenic and analgesic activities [10]. They are also highly significant in agrichemistry, and many of these compounds have been widely used, given their fungicidal [11], insecticidal [12] and herbicidal activities [13].

Pyrazole carboxamide derivatives are important heterocyclic compounds in the development of medicines and pesticides because of their broad spectrum of biological activities, including insecticidal [14], fungicidal [15], herbicidal [13] and acaricidal activity [16]. Many recent studies have been conducted on the synthesis and biological activity of these derivatives. Pyrazole carboxamide derivatives, such as penthiopyrad, furametpyr, penflufen, isopyrazam and bixafen, which could inhibit the succinate dehydrogenase, have been developed and commercialized as fungicides in succession [17]. In addition, many isoxazole compounds, including oxacillin and sulfamethoxazole, have been developed as pesticides and drugs, because of their insecticidal, herbicidal, antiviral and antifungal activities. Isoxazole derivatives have received much attention, because of their wide application in medicine and pesticide chemistry [18].

In view of the facts and to explore the potential antifungal activity of pyrazole derivatives, a series of pyrazole carboxamide and isoxazolol pyrazole carboxylate derivatives are designed and synthesized in the current study. Pyrazole carbonyl chloride is synthesized from pyrazole carboxylic acid and thionyl chloride. Then, 18 novel pyrazole carboxamides and two isoxazolol pyrazole carboxylates are synthesized by reacting pyrazole carbonyl chloride with amines and with isoxazol-3-ol, respectively. The structures of all of the synthesized compounds are unequivocally determined through a comprehensive analysis of spectroscopic data from infrared (IR), mass spectroscopy (MS) and proton nuclear magnetic resonance (^1^H-NMR).

## 2. Results and Discussion

### 2.1. Chemistry

**Scheme 1:**
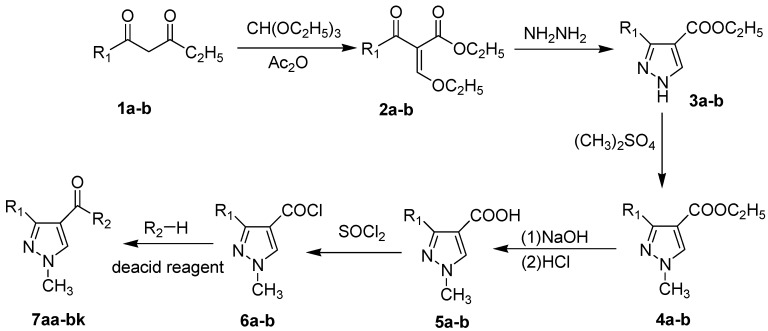
Synthesis route of the pyrazole derivatives.

The synthesis of the derivatives of pyrazole carboxamide and isoxazolol pyrazole carboxylate is outlined in Scheme 1. Acetoacetic ester (**1a**–**b**) and triethyl orthoformate were dissolved in acetic anhydride, refluxed and then converted into 2-ethoxymethylene acetoacetic ester derivatives (**2a**–**b**) [19]. Ethyl 1*H*-pyrazole-4-carboxylate (**3a**–**b**) was prepared by reacting Compounds **2a**–**b** with hydrazine hydrate [20]. Intermediate 1*H*-pyrazole-4-carboxylic acids (**5a**–**b**) were obtained as light-yellow crystals from Compounds **3a**–**b** by successively performing a substitution reaction using dimethyl sulfate, saponification with NaOH and acidification using HCl [21]. Subsequently, Compounds **5a**–**b** were refluxed in SOCl_2_ to yield pyrazole acid chlorides (**6a**–**b**) [22]. Finally, the target compounds of pyrazole carboxamides and isoxazolol pyrazole carboxylates (**7aa**–**bk**) were obtained by reacting **6a**–**b** with different substituted amines [23] and with isoxazol-3-ol, respectively. IR, MS and ^1^H-NMR data were applied to confirm the structures of the new synthesized compounds. The synthesized compounds above were published in our Chinese Patents CN103554026A and CN103524417A [24,25].

### 2.2. Antifungal Activity 

The initial concentration was set at 100 μg/mL for antifungal activity screening *in vitro*. If the percentage inhibition exceeded 50%, a series of concentrations of the compounds was tested to evaluate their EC_50_ values. Carbendazol was selected as the positive control.

As suggested in Table 1, most of the synthesized pyrazole derivatives exhibited antifungal activity to some extent. Though the EC_50_ value of these compounds was higher than that of the positive control, carbendazol, a few pyrazole carboxamides (**7af**, **7bc**, **7bg**, **7bh** and **7bi**) displayed remarkable antifungal activity. According to the literature [17] reported, a bigger group introduced into the ortho position of the aniline part of this type of compound would strengthen the antifungal activity. The simple anilines applied in this study only gave moderate antifungal activity, so the bioactivity and the structures of the pyrazole derivatives should be researched further.

**Table 1:** EC_50_ values of the pyrazole derivatives against four plant pathogenic fungi *in vitro.*

Compound	R_1_	R_2_	EC_50_ (μg/mL)			
			*A. porri*	*M. coronaria*	*C. petroselini*	*R. solani*
**7aa**	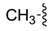	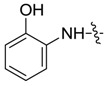	--	--	--	31.39
**7ab**	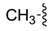	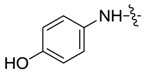	80.76	--	38.41	--
**7ac**	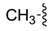	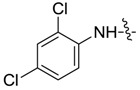	52.56	84.74	--	40.00
**7ad**	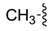	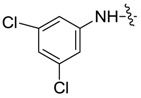	--	--	6.32	18.15
**7ae**	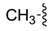	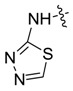	65.12	--	--	14.89
**7af**	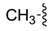	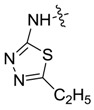	63.04	7.87	35.90	5.23
**7ag**	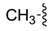	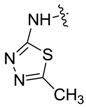	54.86	76.12	51.00	16.91
**7ah**	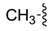	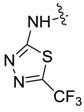	--	--	--	69.45
**7ai**	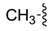	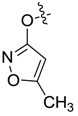	2.24	3.21	10.19	0.37
**7ba**	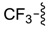	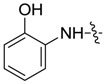	--	35.94	22.47	16.81
**7bb**	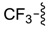	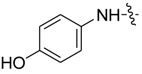	--	74.54	27.82	19.47
**7bc**	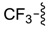	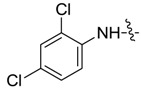	10.10	14.92	5.43	3.40
**7bd**	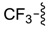	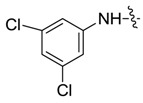	--	--	74.38	27.37
**7be**	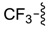	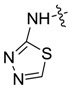	23.12	13.00	13.44	8.55
**7bf**	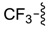	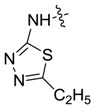	72.20	61.29	--	81.72
**7bg**	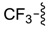	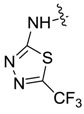	11.22	7.93	27.43	4.99
**7bh**	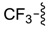	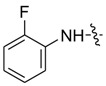	24.76	25.48	6.99	5.93
**7bi**	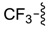	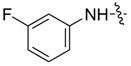	21.01	9.08	32.40	7.69
**7bj**	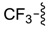	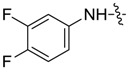	11.46	15.86	--	8.32
**7bk**	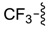	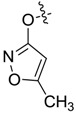	35.05	--	--	28.88
**carbendazol**			0.99	0.96	0.96	1.00

“--” The percentage of inhibition is lower than 50% at 100 μg/mL.

It was interesting that the isoxazolol pyrazole carboxylate 7**ai** showed significant antifungal activity against *A. porri*, *M. coronaria*, *C. petroselini* and *R. solani*, with EC_50_ values of 2.24, 3.21, 10.29 and 0.37 μg/mL, respectively. The EC_50_ value of this compound against *R. solani* was lower than that of the positive control, carbendazol (EC_50_, 1.00 μg/mL).

The EC_50_ values of the isoxazolol pyrazole carboxylate **7bk** were 35.05, over 100, over 100 and 28.88 μg/mL against *A. porri*, *M. coronaria*, *C. petroselini* and *R. solani*, respectively (Table 1). The results of the preliminary structure-activity relationship (SAR) analysis suggested that the antifungal activity of the synthesized isoxazolol pyrazole carboxylate was significantly weakened when the methyl group at the C-3 of the pyrazole ring (**7ai**) was substituted with a trifluoromethyl group (**7bk**).

## 3. Experimental Section

### 3.1. Chemistry

All of the reagents and solvents were either chemically pure or purified in accordance with standard methods. Reactions were monitored through thin-layer chromatography (TLC) using precoated silica gel plates (silica gel GF 254, Qingdao Marine Chemistry Co. Ltd., Qingdao, China), and the spots were visualized with UV (254 nm). All of the melting points were detected with a WRS-1A type melting point apparatus (ShangHai Suoguang Electric Tech Co., Ltd., Shanghai, China), and the thermometer was not corrected. IR spectra were recorded on a Nicolet IR-200 (Thermo Electron, Madison, WI, USA) spectrophotometer. ^1^H-NMR spectra were captured with Bruker AV-500 and AV-400 spectrometers, and tetramethylsilane was applied as an internal standard. High-resolution electrospray ionization mass spectroscopy (HR-ESI-MS) spectra were observed with a Micromass Q-TOF spectrometer (Waters Corp., Manchester, UK).

### 3.2. General Procedure for the Preparation of ***2a**–**b***

A mixture of acetoacetic ester (0.60 mol) (**1a**–**b**), triethyl orthoformate (0.72 mol) and acetic anhydride (1.08 mol) was stirred and heated under reflux until the **1a**–**b** was no longer monitored by TLC. Then, the reaction mixture was evaporated *in vacuo.* The distillates of T = 140 °C–160 °C (3 KPa) were collected to produce 2-ethoxymethylene acetoacetic esters (**2a**–**b**) as a light-yellow liquid with yields ranging from 70% to 90%.

### 3.3. General Procedure for the Preparation of ***3a**–**b***

The acetoacetic ester of 2-ethoxymethylene (0.2 mol) (**2a**–**b**) was dissolved in ethanol (150 mL) in an ice-water bath, and 80% hydrazine hydrate (0.4 mol) was added dropwise. The mixture reacted at room temperature until the **2a**–**b** was fully consumed, as detected by TLC. Subsequently, the reaction mixture was concentrated *in vacuo.* The residue was extracted with 1,2-dichloroethane, washed with water and brine, dried over anhydrous sodium sulfate and then concentrated *in vacuo* to obtain ethyl 1*H*-pyrazole-4-carboxylate (**3a**–**b**) as either light-yellow liquids or solids.

### 3.4. General Procedure for the Preparation of ***5a**–**b***

Ethyl 1*H*-pyrazole-4-carboxylate (**3a**–**b**, 0.5 mol) and NaHCO_3_ (0.6 mol) were dissolved in toluene (120 mL). (CH_3_)_2_SO_4_ (0.24 mol) was dropped gradually into the solution while the temperature was maintained at 20 °C–30 °C via an ice-water bath. Then, the solution reacted at a temperature of 50 °C with a water bath and was monitored by TLC. Once the reaction was complete, the reaction solution was filtered. A light-yellow solution was then obtained and washed with ice water. The upper toluene solution was concentrated *in vacuo* to produce ethyl 1-methyl-1*H*-pyrazole-4-carboxylate (**4a**–**b**). Subsequently, sodium hydroxide solution (0.12 mol NaOH dissolved in 45 mL water) was added to a solution of Compounds **4a**–**b** (0.1 mol) in EtOH (95%, 50 mL) and then reacted at room temperature for approximately 2 h. The solution was concentrated *in vacuo* to remove most of the ethanol. The pH level was then adjusted to 3–4 with HCl. The reaction mixture was filtered, and the filtrate was recrystallized with ethyl acetate to produce pyrazole acids (**5a**–**b**) as light-yellow crystalline solids.

### 3.5. General Procedure for the Preparation of ***7aa**–**bk***

Pyrazole acid chlorides **6a**–**b** were prepared by refluxing **4a**–**b** in thionyl chloride for 8 h. Pyrazole acid chlorides **6a**–**b** (12 mmol) in anhydrous tetrahydrofuran (THF; 30 mL) were slowly added to a solution of amine derivatives or 5-methylisoxazol-3-ol (10 mmol) and K_2_CO_3_ (1.38 g, 10 mmol) in anhydrous THF (30 mL) at a controlled temperature of 5 °C. The reaction proceeded at room temperature until **6a**–**b** was no longer tested by TLC. The reaction solution was then filtered and the solvent distilled. The residue was dissolved in ethyl acetate, washed with water and brine, dried over anhydrous sodium sulfate and recrystallized to generate the target pyrazole carboxamides and isoxazolol pyrazole carboxylates (**7aa**–**bk)**. The product yields ranged from 40% to 80%. All 20 compounds were novel, and the physical and spectral data for these compounds are listed below.

*1,3-Dimethyl-N-(2-hydroxyl)benzyl-1H-pyrazole-4-carboxamide* (**7aa**): Bright brown crystal, yield of 77.9%, m.p. 193.0–193.1 °C. ^1^H-NMR (CDCl_3_, 500 MHz) δ: 8.82 (s, 1H, NH), 7.86 (s, 1H, pyrazole H), 7.61 (s, 1H, -OH), 7.14–7.16 (m, 1H, Ar-H), 7.13–7.09 (m, 1H, Ar-H), 7.08–7.05 (m, 1H, Ar-H), 6.89 (t, *J* = 1.5 Hz, 1H, Ar-H), 3.89 (s, 3H, N-CH_3_), 2.56 (s, 3H, pyrazole CH_3_); IR (KBr): ν 3431, 1643, 1593, 1544, 1452, 1382, 1278 cm^−1^; HR-ESI-MS *m*/*z*: 232.1084 [M+H]^+^ (calcd. for C_12_H_1__4_N_3_O_2_, 232.1081).

*1,3-Dimethyl-N-(4-hydroxyl)benzyl-1H-pyrazole-4-carboxamide* (**7ab**): Gray needle crystal, yield of 80.5%, m.p. 208.0–208.7 °C. ^1^H-NMR (CDCl_3_, 500 MHz) δ: 7.76 (s, 1H, pyrazole H), 7.43 (m, 2H, Ar-H), 6.84 (d, *J* = 8.5 Hz, 2H, Ar-H), 4.78 (s, 1H, -OH), 3.89 (s, 3H, N-CH_3_), 2.54 (s, 3H, pyrazole CH_3_); IR (KBr): ν 3510, 3290, 1639, 1539, 1515, 1448, 1245, 1166 cm^−1^; HR-ESI-MS *m*/*z*: 232.1081 [M+H]^+^ (calcd. for C_12_H_1__4_N_3_O_2_, 232.1081). 

*1,3-Dimethyl-N-2',4'-dichlorobenzyl-1H-pyrazole-4-carboxamide* (**7ac**): White lamellar crystal, yield of 77.9%, m.p. 169.1–169.3 °C. ^1^H-NMR (CDCl_3_, 500 MHz) δ: 8.52 (d, *J* = 9.0 Hz, 1H, Ar-H), 7.86 (s, 1H, pyrazole H), 7.42 (d, *J* = 2.5 Hz, 1H, Ar-H), 7.27–7.30 (m, 1H, Ar-H), 3.91 (s, 3H, N-CH_3_), 2.60 (s, 3H, pyrazole CH_3_); IR (KBr): ν 3241, 1648, 1499, 1279, 1096 cm^−1^; HR-ESI-MS *m*/*z*: 284.0347 [M+H]^+^ (calcd. for C_12_H_12_Cl_2_N_3_O, 284.0352).

*1,3-Dimethyl-N-3',5'-dichlorobenzyl-1H-pyrazole-4-carboxamide* (**7ad**): Gray crystal, yield of 66.9%, m.p. 181.6–182.2 °C. ^1^H-NMR (CDCl_3_, 500 MHz) δ: 7.77 (s, 1H, pyrazole H), 7.54 (d, *J* = 1.5 Hz, 2H, Ar-H), 7.11 (s, 1H, Ar-H), 3.88 (s, 3H, N-CH_3_), 2.53 (s, 3H, pyrazole CH_3_); IR (KBr): ν 3265, 3211, 1698, 1614, 1503, 1403, 1142, 1063 cm^−1^; HR-ESI-MS *m*/*z*: 284.0357 [M+H]^+^ (calcd. for C_12_H_12_Cl_2_N_3_O, 284.0352).

*1,3-Dimethyl-N-(1,3,4-thiadiazole-2-yl)-1H-pyrazole-4-carboxamide* (**7ae**): White lamellar crystal, yield of 57.2%, m.p. 278.4–278.9 °C. ^1^H-NMR (DMSO-*d*_6_, 500 MHz) δ: 12.52 (s, 1H, NH), 9.15 (s, 1H, pyrazole H), 8.55 (s, 1H, thiadiazole H), 3.82 (s, 3H, N-CH_3_), 2.50 (s, 3H, pyrazole CH_3_); IR (KBr): ν 3381, 3124, 2929, 1680, 1548, 1415, 1311 cm^−1^; HR-ESI-MS *m*/*z*: 224.0597 [M+H]^+^ (calcd. for C_8_H_10_N_5_OS, 224.0601).

*1,3-Dimethyl-N-(5-ethyl-1,3,4-thiadiazole-2-yl)-1H-pyrazole-4-carboxamide* (**7af**): White powder, yield of 68.3%, m.p. 266.2–266.5 °C. ^1^H-NMR (CDCl_3_, 400 MHz) δ: 12.58 (s, 1H, NH), 9.13 (s, 1H, pyrazole H), 3.94 (s, 3H, N-CH_3_), 3.07 (q, *J* = 1.0 Hz, 2H, CH_2_), 2.56 (s, 3H, pyrazole CH_3_), 1.45 (t, *J* = 1.0 Hz, 3H, CH_3_); IR (KBr): ν 3129, 2971, 2934, 1673, 1544, 1420, 1316, 1241, 1179 cm^−1^; HR-ESI-MS *m*/*z*: 252.0912 [M+H]^+^ (calcd. for C_10_H_14_N_5_OS, 252.0914).

*1,3-Dimethyl-N-(5-methyl-1,3,4-thiadiazole-2-yl)-1H-pyrazole-4-carboxamide* (**7ag**): White powder, yield of 61.4%, m.p. >300 °C. ^1^H-NMR (CDCl_3_, 400 MHz) δ: 9.12 (s, 1H, pyrazole H), 3.96 (s, 3H, N-CH_3_), 2.72 (s, 3H, thiadiazole CH_3_), 2.56 (s, 3H, pyrazole CH_3_); IR (KBr): ν 3149, 3012, 2921, 1677, 1544, 1494, 1416, 1320, 1250, 1188 cm^−1^; HR-ESI-MS *m*/*z*: 238.0755 [M+H]^+^ (calcd. for C_9_H_1__2_N_5_OS, 238.0757).

*1,3-Dimethyl-N-(5-trifluoromethyl-1,3,4-thiadiazole-2-yl)-1H-pyrazole-4-carboxamide* (**7ah**): White lamellar crystal, yield of 44.3%, m.p. 272.1–272.8 °C. ^1^H-NMR (CDCl_3_, 500 MHz) δ: 12.61 (s, 1H, NH), 9.12 (s, 1H, pyrazole H), 3.98 (s, 3H, N-CH_3_), 2.58 (s, 3H, pyrazole CH_3_); IR (KBr): ν 1668, 1519, 1303, 1195, 1137, 1029 cm^−1^; HR-ESI-MS *m*/*z*: 292.0471 [M+H]^+^ (calcd. for C_9_H_9_F_3_N_5_OS, 292.0474).

*5-Methylisoxazol-3-yl 1,3-dimethyl-1H-pyrazole-4-carboxylate* (**7ai**): White powder, yield of 54.7%, m.p. 124.2–124.4 °C. ^1^H-NMR (CDCl_3_, 500 MHz) δ: 7.98 (s, 1H, pyrazole H), 6.23 (s,1H, isoxazole H), 3.88 (s, 3H, N-CH_3_), 2.49 (s, 3H, pyrazole CH_3_), 2.43 (s, 3H, isoxazole CH_3_); IR (KBr): ν 3120, 1743, 1622, 1548, 1232, 1050 cm^−1^; HR-ESI-MS *m*/*z*: 222.0871 [M+H]^+^ (calcd. for C_10_H_12_N_3_O_3_, 222.0873).

*1-Methyl-N-(2-hydroxyl)benzyl-3-(trifluoromethyl)-1H-pyrazole-4-carboxamide* (**7ba**): Light yellow crystal, yield of 41.8%, m.p. 192.8–193.2 °C. ^1^H-NMR (DMSO-*d*_6_, 500 MHz) δ: 9.24 (s, 1H, -OH), 8.54 (s, 1H, pyrazole H), 7.65 (d, *J* = 7.5 Hz, 1H, Ar-H), 6.98 (d, *J* = 7.5 Hz, 1H, Ar-H), 6.88 (d, *J* = 7.5 Hz, 1H, Ar-H), 6.79 (d, *J* = 8.0 Hz, 1H, Ar-H), 3.94 (s, 3H, N-CH_3_); IR (KBr): ν 3485, 3116, 1656, 1598, 1494, 1441, 1329, 1250, 1175, 1146 cm^−1^; HR-ESI-MS *m*/*z*: 286.0804 [M+H]^+^ (calcd. for C_12_H_11_F_3_N_3_O_2_, 286.0798).

*1-Methyl-N-(4-hydroxyl)benzyl-3-(trifluoromethyl)-1H-pyrazole-4-carboxamide* (**7bb**): light brown powder, yield of 67.9%, m.p. 206.4–206.6 °C. ^1^H-NMR (DMSO-*d*_6_, 500 MHz) δ: 9.88 (s, 1H, NH), 9.25 (s, 1H, -OH), 8.44 (s, 1H, pyrazole H), 7.38–7.43 (m, 2H, Ar-H), 6.69–6.72 (m, 2H, Ar-H), 3.98 (s, 3H, N-CH_3_); IR (KBr): ν 1627, 1553, 1436, 1329, 1142, 839, 781, 711, 619, 516 cm^−1^; HR-ESI-MS *m*/*z*: 286.0800 [M+H]^+^ (calcd. for C_12_H_11_F_3_N_3_O_2_, 286.0798).

*1-Methyl-N-2',4'-dichlorobenzyl-3-(trifluoromethyl)-1H-pyrazole-4-carboxamide* (**7bc**): White crystal, yield of 49.0%, m.p. 147.4–147.8 °C. ^1^H-NMR (CDCl_3_, 400 MHz) δ: 8.43 (d, *J* = 7.2 Hz, 1H, Ar-H), 8.04 (s, 1H, pyrazole H), 7.43 (d, *J* = 2.0 Hz, 1H, Ar-H), 7.26–7.30 (m, overlapped, Ar-H), 4.02 (s, 3H, N-CH_3_); IR (KBr): ν 2958, 1785, 1640, 1565, 1424, 1333, 1291, 1146, 1047, 847, 781, 706 cm^−1^; HR-ESI-MS *m*/*z*: 338.0066 [M+H]^+^ (calcd. for C_12_H_9_Cl_2_F_3_N_3_O, 338.0069). 

*1-Methyl-N-3',5'-dichlorobenzyl-3-(trifluoromethyl)-1H-pyrazole-4-carboxamide* (**7bd**): White powder, yield of 53.2%, m.p. 149.1–150.3 °C. ^1^H-NMR (CDCl_3_, 500 MHz) δ: 8.42 (d, *J* = 9.0 Hz, Ar-H), 8.03 (s, 1H, pyrazole H), 7.42 (d, *J* = 2.0 Hz, 1H, Ar-H), 7.26–7.30 (m, overlapped, Ar-H), 4.01 (s, 3H, N-CH_3_); IR (KBr): ν 3299, 3162, 3100, 1669, 1582, 1523, 1494, 1461, 1387, 1304 cm^−1^; HR-ESI-MS *m*/*z*: 338.0075 [M+H]^+^ (calcd. for C_12_H_9_Cl_2_F_3_N_3_O, 338.0069). 

*1-Methyl-N-(1,3,4-thiadiazole-2-yl)-3-(trifluoromethyl)-1H-pyrazole-4-carboxamide* (**7be**): White floccus, yield of 49.6%, m.p. >300 °C. ^1^H-NMR (DMSO-*d*_6_, 500 MHz) δ: 12.99 (s, 1H, NH), 9.21 (s, 1H, thiadiazole H), 8.76 (s, 1H, pyrazole H), 3.99 (s, 3H, N-CH_3_); IR (KBr): ν 3170, 3116, 3054, 1689, 1557, 1436, 1316, 1175, 1134, 1059, 1005, 872 cm^−1^; HR-ESI-MS *m*/*z*: 278.0320 [M+H]^+^ (calcd. for C_8_H_7_F_3_N_5_OS, 278.0318). 

*1-Methyl-N-(5-ethyl-1,3,4-thiadiazole-2-yl)-3-(trifluoromethyl)-1H-pyrazole-4-carboxamide* (**7bf**): White powder, yield of 56.9%, m.p. 288.6–288.8 °C. ^1^H-NMR (DMSO-*d*_6_, 500 MHz) δ: 12.82 (s, 1H, NH), 8.72 (s, 1H, pyrazole H), 3.96 (s, 3H, N-CH_3_), 2.98 (q, *J* = 7.5 Hz, 2H, CH_2_), 1.29 (t, *J* = 7.5 Hz, 3H, CH_3_); IR (KBr): ν 3456, 3054, 2963, 1694, 1627, 1557, 1424, 1341, 1308, 1171, 1129 cm^−1^; HR-ESI-MS *m*/*z*: 306.0631 [M+H]^+^ (calcd. for C_10_H_11_F_3_N_5_OS, 306.0631).

*1-Methyl-N-(5-trifluoromethyl-1,3,4-thiadiazole-2-yl)-3-(trifluoromethyl)-1H-pyrazole-4-carboxamide* (**7bg**): White crystal, yield of 71.2%, m.p. 234.9–235.1 °C. ^1^H-NMR (DMSO-*d*_6_, 500 MHz) δ: 13.68 (s, 1H, NH), 8.81 (s, 1H, pyrazole H), 4.01 (s, 3H, N-CH_3_); IR (KBr): ν 3456, 3070, 2963, 1694, 1627, 1557, 1424, 1341, 1308, 1183, 885, 711 cm^−1^; HR-ESI-MS *m*/*z*: 346.0191 [M+H]^+^ (calcd. for C_9_H_6_F_6_N_5_OS, 346.0192). 

*1-Methyl-N-2'-fluorobenzyl-3-(trifluoromethyl)-1H-pyrazole-4-carboxamide* (**7bh**): Brown crystal, yield of 74.3%, m.p. 148.4~148.9 °C. ^1^H-NMR (CDCl_3_, 500 MHz) δ: 8.38 (m, 1H, Ar-H), 8.03 (s, 1H, pyrazole H), 8.00–8.03 (m, 1H, Ar-H), 7.08–7.18 (m, 2H, Ar-H), 4.00 (s, 3H, N-CH_3_); IR (KBr): ν 3245, 1652, 1565, 1507, 1441, 1316, 1217, 1183 cm^−1^; HR-ESI-MS *m*/*z*: 288.0757 [M+H]^+^ (calcd. for C_12_H_10_F_4_N_3_O, 288.0755).

*1-Methyl-N-3'-fluorobenzyl-3-(trifluoromethyl)-1H-pyrazole-4-carboxamide* (**7bi**): Brown crystal, yield of 47.9%, m.p. 137.8–138.3 °C. ^1^H-NMR (CDCl_3_, 500 MHz) δ: 8.04 (s, 1H, pyrazole H), 7.76 (s, 1H, Ar-H), 7.58 (d, *J* = 10.5 Hz, 1H, Ar-H), 7.18 (d, *J* = 8.5 Hz, 1H, Ar-H), 6.87 (t, *J* = 8.5 Hz, 1H, Ar-H), 4.01 (s, 3H, N-CH_3_); IR (KBr): ν 3494, 3373, 3261, 1664, 1602, 1561, 1329, 1300, 1188 cm^−1^; HR-ESI-MS *m*/*z*: 288.0757 [M+H]^+^ (calcd. for C_12_H_10_F_4_N_3_O, 288.0755). 

*1-Methyl-N-3',4'-difluorobenzyl-3-(trifluoromethyl)-1H-pyrazole-4-carboxamide* (**7bj**): Light brown crystal, yield of 63.2%, m.p. 155.7–155.8 °C. ^1^H-NMR (CDCl_3_, 400 MHz) δ: 8.04 (s, 1H, pyrazole H), 7.78 (s, 1H, Ar-H), 7.69 (d, *J* = 7.5 Hz, 1H, Ar-H), 7.15 (d, *J* = 7.0 Hz, 1H, Ar-H), 4.00 (s, 3H, N-CH_3_); IR (KBr): ν 3241, 1652, 1557, 1519, 1432, 1329, 1300, 1208, 1146, 1051, 827, 752 cm^−1^; HR-ESI-MS *m*/*z*: 306.0663 [M+H]^+^ (calcd. for C_12_H_9_F_5_N_3_O, 306.0660).

*5-Methylisoxazol-3-yl 1-methyl-3-(trifluoromethyl)-1H-pyrazole-4-carboxylate* (**7bk**): White powder, yield of 58.9%, m.p. 130.7–131.1 °C. ^1^H-NMR (CDCl_3_, 400 MHz) δ: 8.19 (s, 1H, pyrazole H), 6.28 (s, 1H, isoxazole H), 4.03 (s, 3H, N-CH_3_), 2.45 (s, 3H, isoxazole CH_3_); IR (KBr): ν 3290, 1660, 1561, 1523, 1436, 1300, 1134, 1059, 856, 823, 711, 648 cm^−1^; HR-ESI-MS *m*/*z*: 276.0595 [M+H]^+^ (calcd. for C_10_H_9_F_3_N_3_O_3_, 276.0591). 

### 3.6. Antifungal Bioassays

The fungicidal activity of the target Compounds **7aa**–**bk** were tested *in vitro* against the phytopathogenic fungi *A. porri*, *M. coronaria*, *C. petroselini* and *R. solani* using the mycelium growth rate method [26,27]. The commercially available agricultural fungicide, carbendazol, was used as the positive control, whereas acetone served as the negative control. The compounds were dissolved in acetone to prepare a 100-μg/mL stock solution for the following antifungal test.

After the mycelia were incubated at 25 °C over a certain period, the diameter of each strain was measured. The percentage inhibition was calculated as follows:

*I* = (*B* − *A*)/*B* × 100%

where *I* is the percentage of inhibition, *A* is the average mycelia diameter (mm) with the compounds in Petri dishes and *B* is the average mycelia diameter with the compounds in the blank Petri dishes. 

The percentage inhibition of the compounds was determined at a dosage of 100 μg/mL. The compounds that displayed high activity (*I* > 50% at 100 μg/mL) were evaluated further at concentrations of 100, 50, 25, 12.5, 6.25 and 0 μg/mL. Three replicates were applied in each treatment. The EC_50_ (μg/mL) values were estimated statistically by Probit analysis using SPSS (version 11.5) on a personal computer. 

## 4. Conclusions

In conclusion, a series of novel pyrazole carboxamides and isoxazolol pyrazole carboxylates was synthesized and characterized based on the spectral data of ^1^H-NMR, IR and MS in this study. The antifungal activity of the compounds was evaluated *in vitro* against the phytopathogenic fungi *A. porri*, *M. coronaria*, *C. petroselini* and *R. solani*. Among these compounds, the pyrazole carboxamides **7af**, **7bc**, **7bg**, **7bh** and **7bi** exhibited moderate antifungal activity. The isoxazolol pyrazole carboxylate **7ai** displayed strong antifungal activity against *R. solani*, with an EC_50_ value of 0.37 μg/mL. This value was better than that of the commercial fungicide, carbendazol. SAR analysis results suggested that the antifungal activity of the synthesized isoxazolol pyrazole carboxylate was significantly weakened when the methyl at the C-3 of the pyrazole ring was substituted with trifluoromethyl. Thus, these novel antifungal molecules can be considered promising lead compounds with which to explore biological activity in future research.

## Supplementary Materials

Supplementary materials can be accessed at: http://www.mdpi.com/1420-3049/20/03/4383/s1.
