# Supplementary material for: Synthesis and Antifungal Activity of the Derivatives of Novel Pyrazole Carboxamide and Isoxazolol Pyrazole Carboxylate
Source: Molecules. 2015 Mar 9;20(3):4383–94. doi: 10.3390/molecules20034383 (PMC6272414; doi:10.3390/molecules20034383)
Supplement: Supplementary file 1 [file molecules-20-04383-s001.pdf]

Supplementary Materials

The ^{1}H-NMR, IR and MS Spectra of Compounds **7aa–bk**

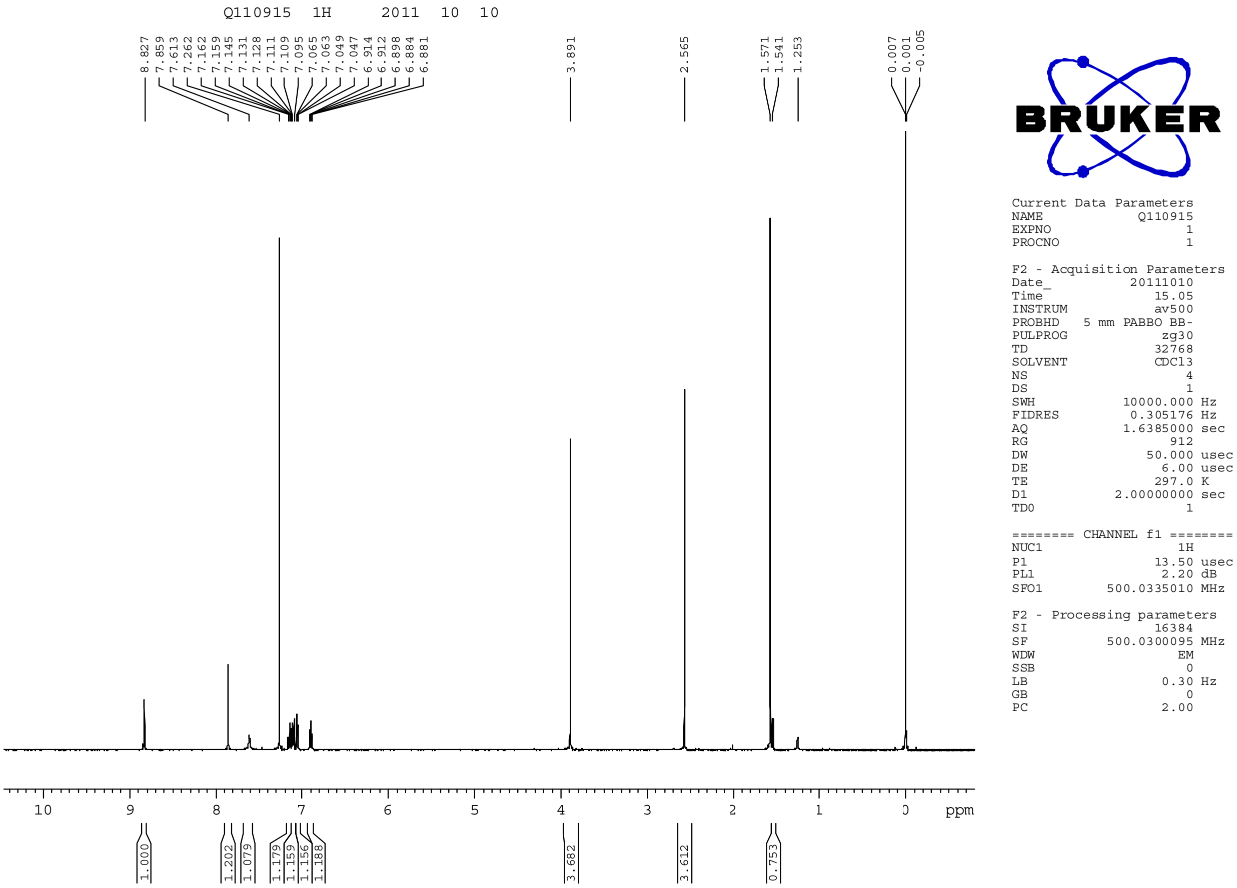

Figure S1. The ^{1}H-NMR (CDCl$_3$, 500 MHz) spectra of compound **7aa**.

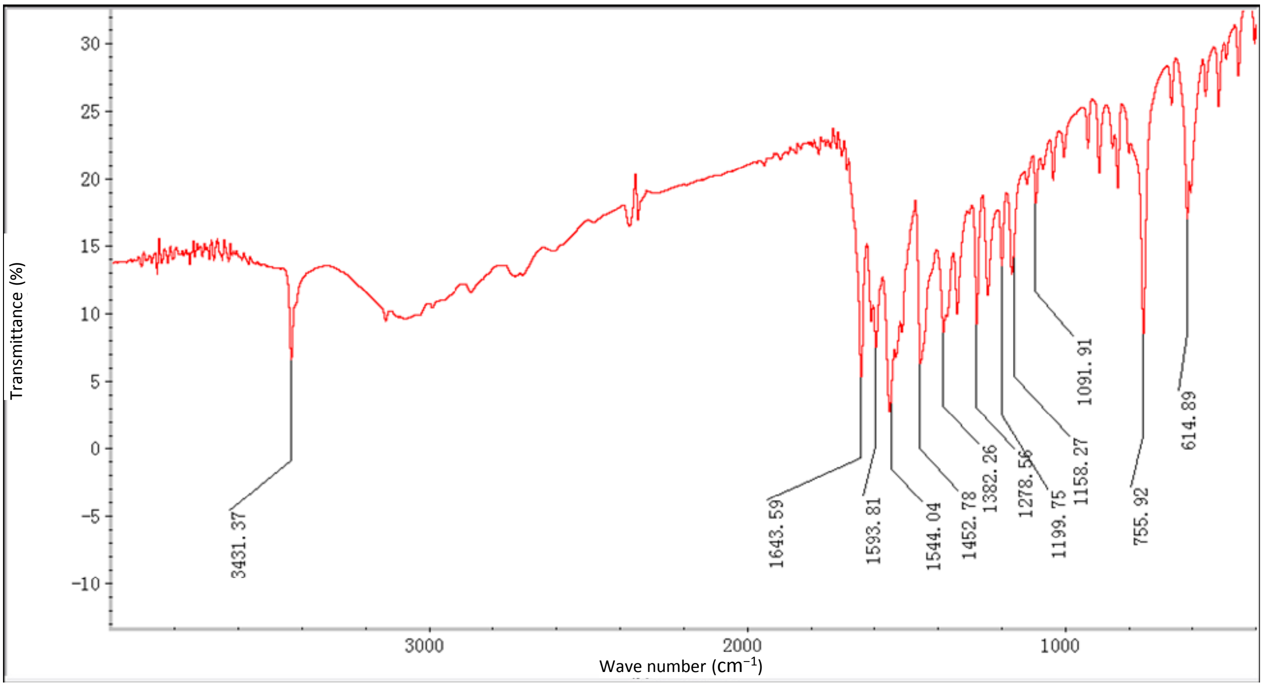

Figure S2. The IR spectra of compound **7aa**.

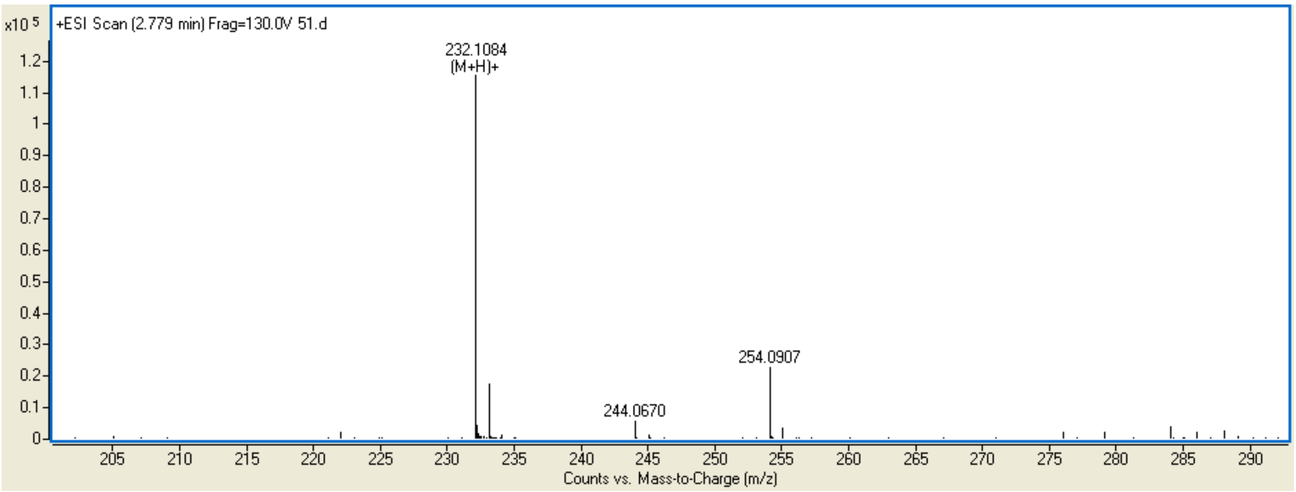

Figure S3. The HR-ESI-MS of compound **7aa**.

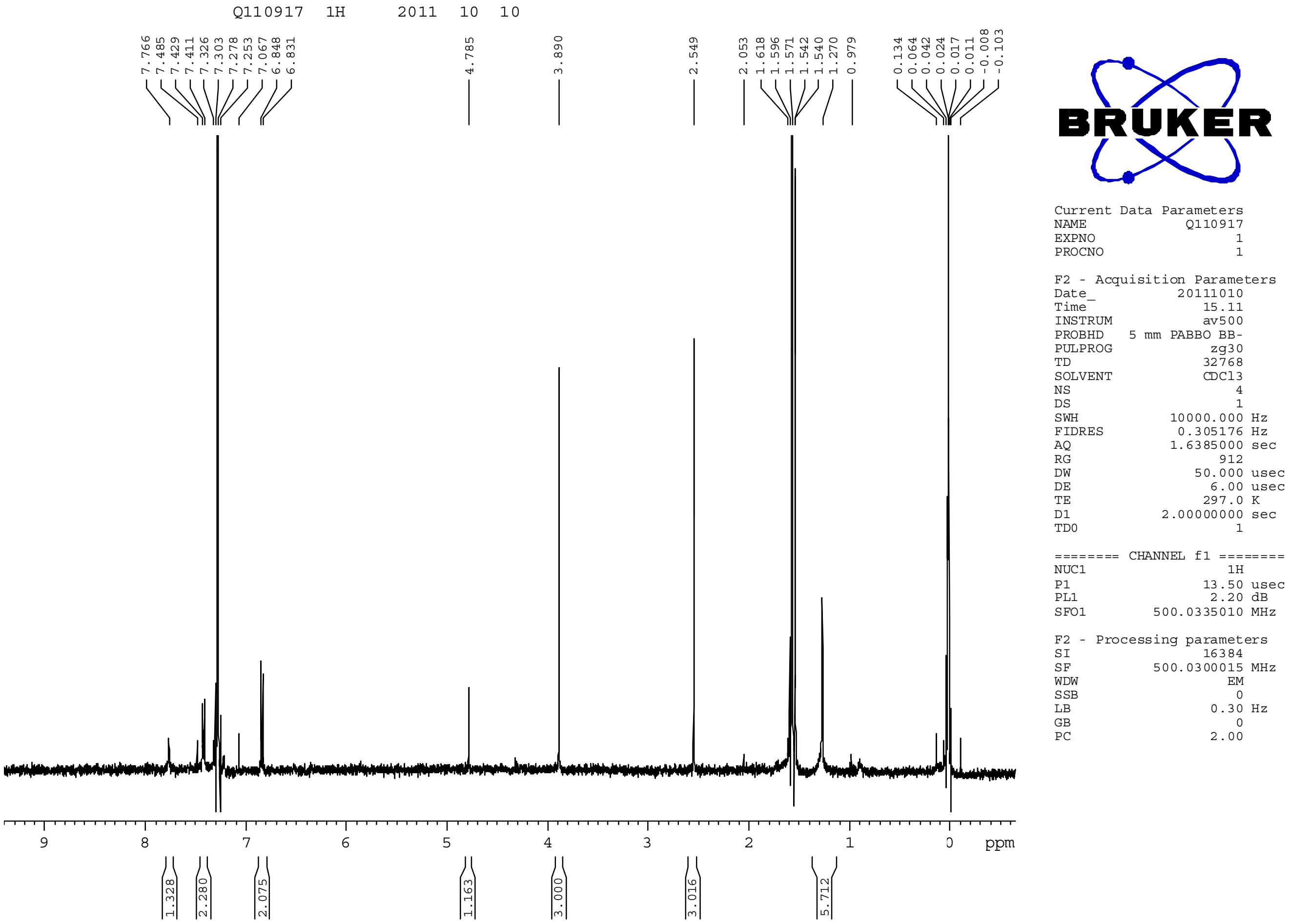

Figure S4. The ^{1}H-NMR ($CDCl_3$, 500 MHz) spectra of compound **7ab**.

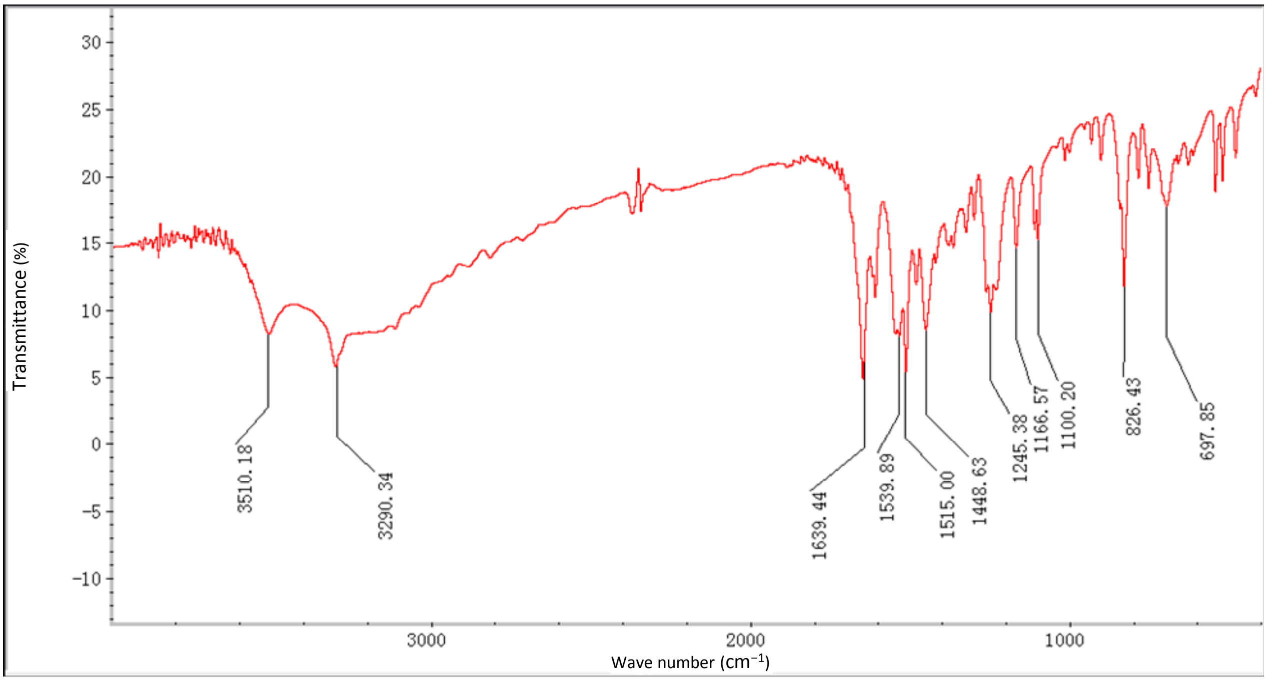

Figure S5. The IR spectra of compound **7ab**.

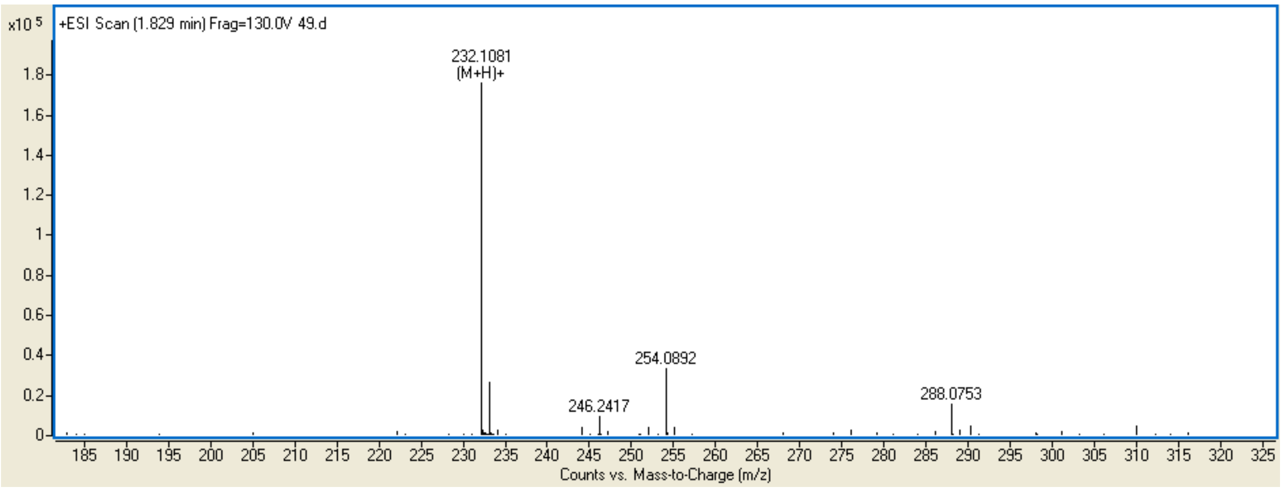

Figure S6. The HR-ESI-MS of compound **7ab**.

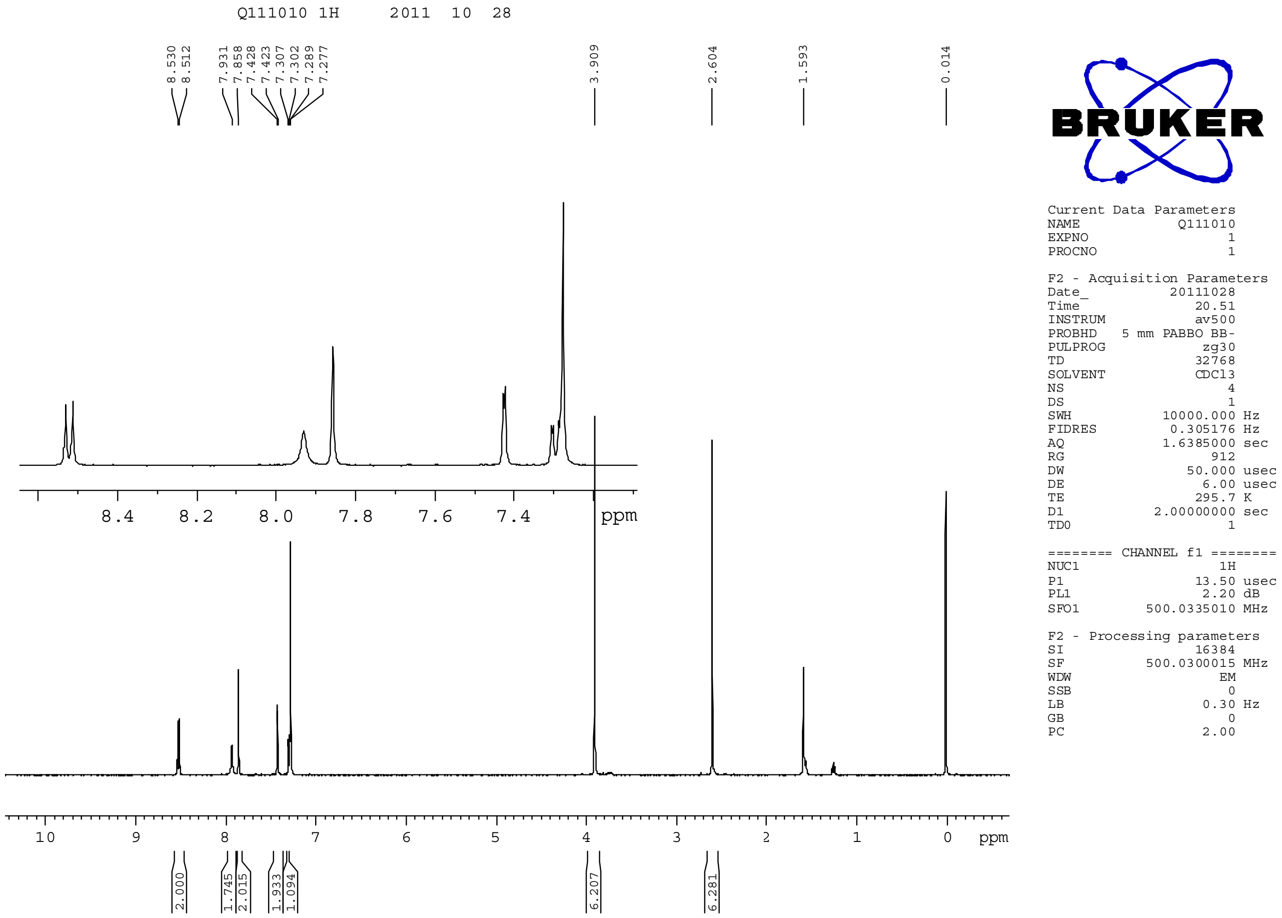

Figure S7. The ^{1}H-NMR ($CDCl_3$, 500 MHz) spectra of compound **7ac**.

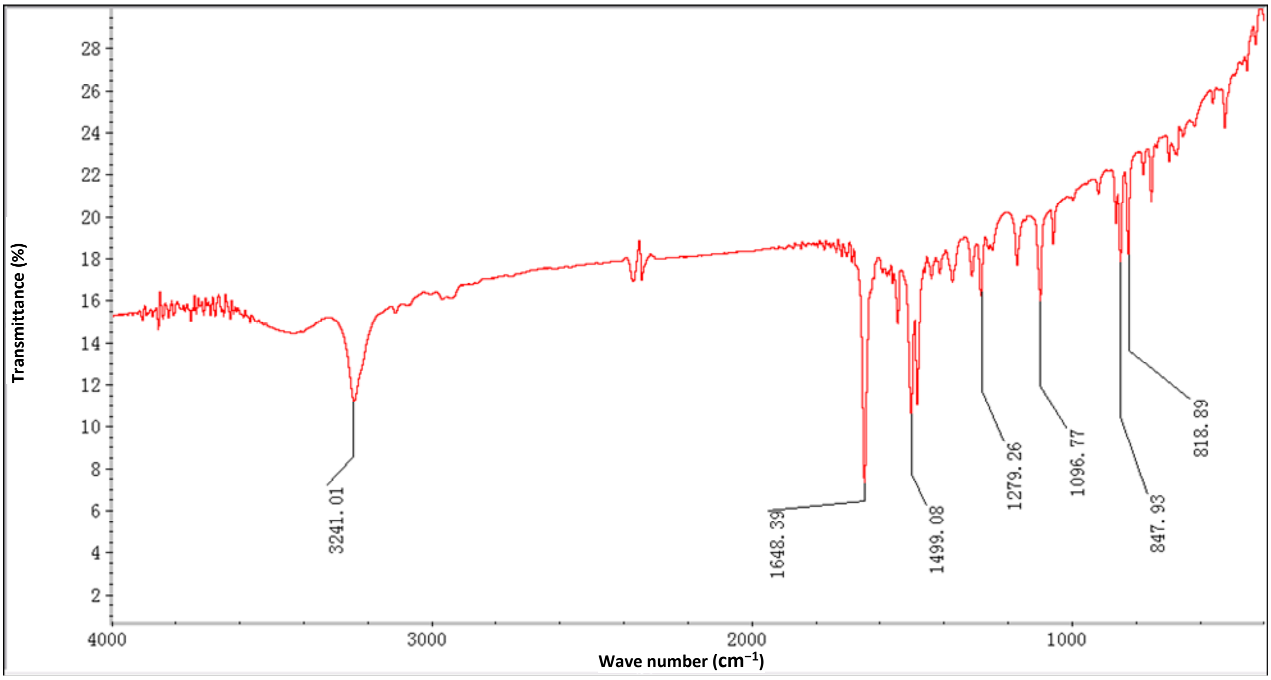

Figure S8. The IR spectra of compound **7ac**.

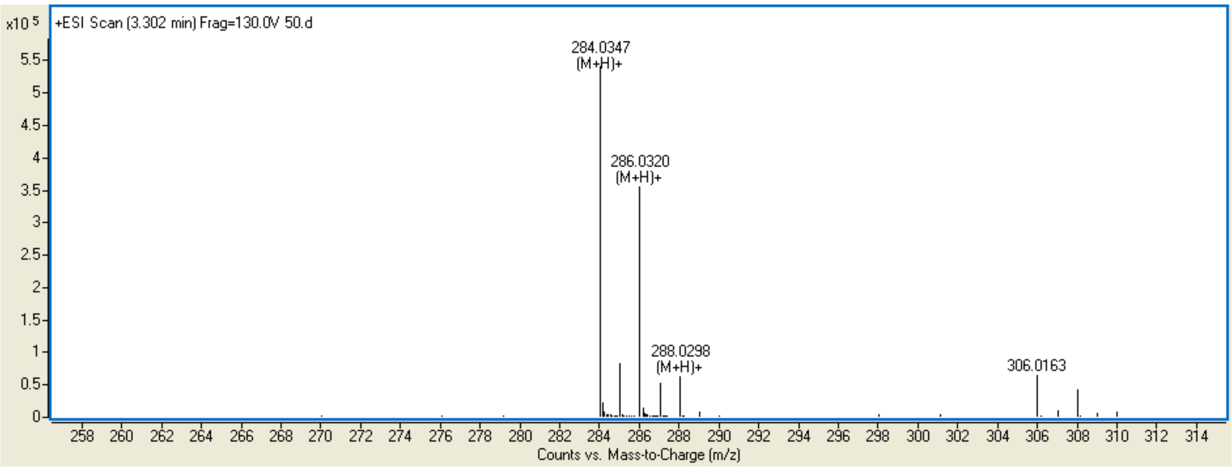

Figure S9. The HR-ESI-MS of compound **7ac**.

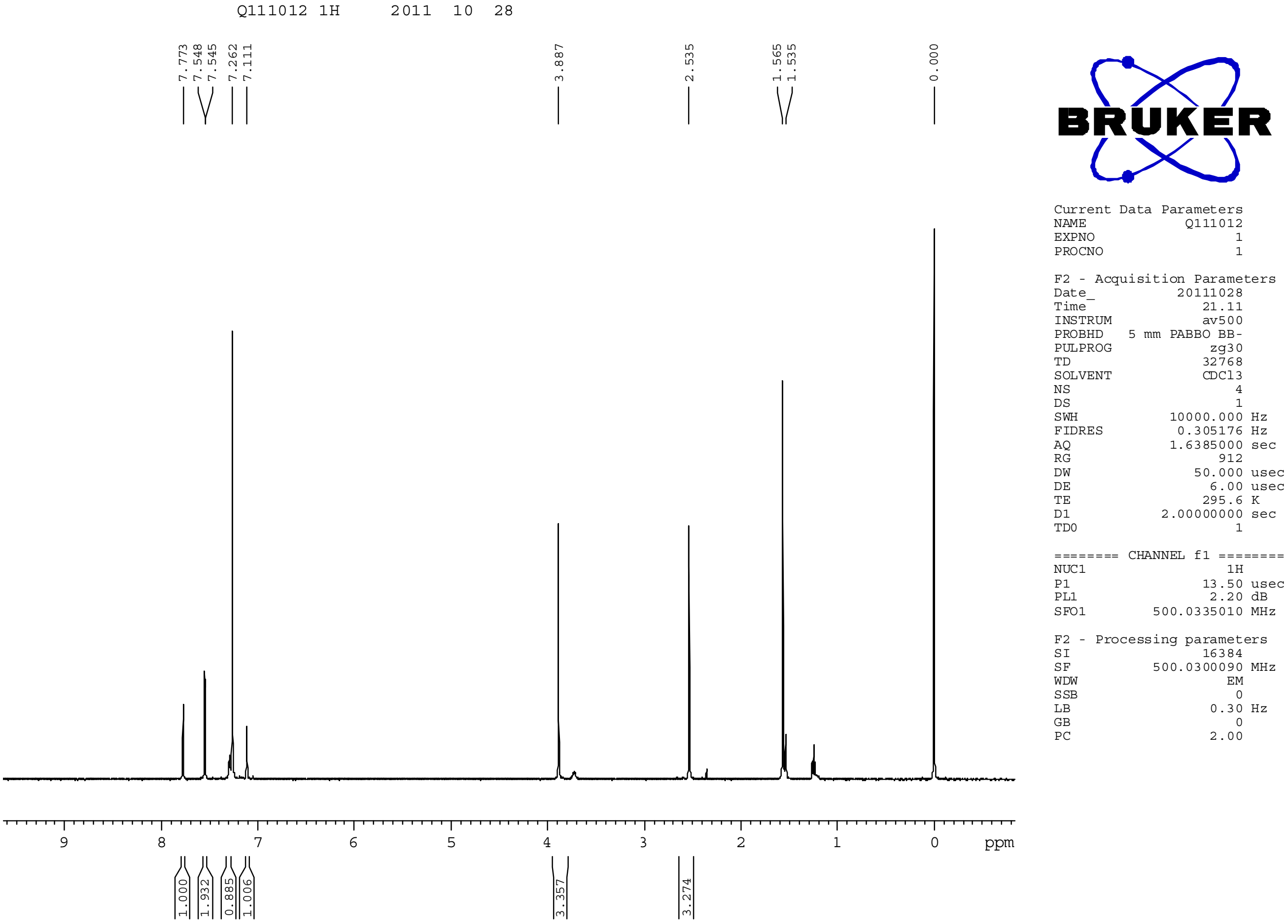

Figure S10. The ^{1}H-NMR ($CDCl_3$, 500 MHz) spectra of compound **7ad**.

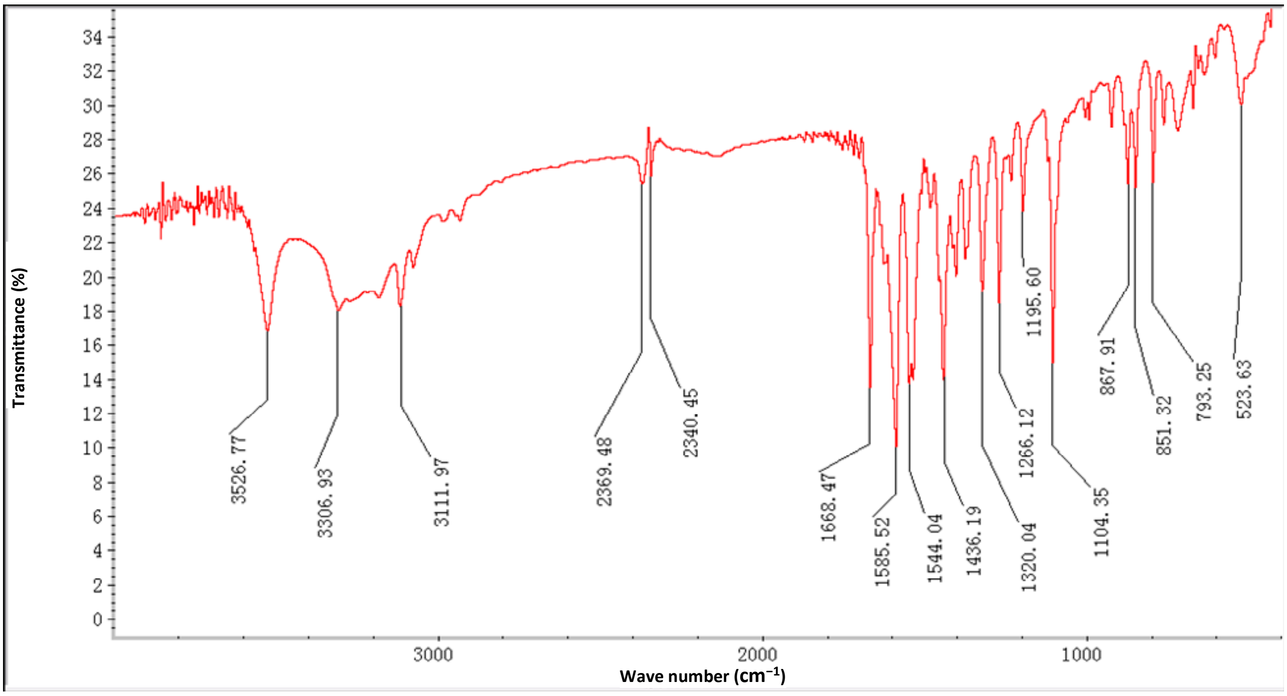

Figure S11. The IR spectra of compound **7ad**.

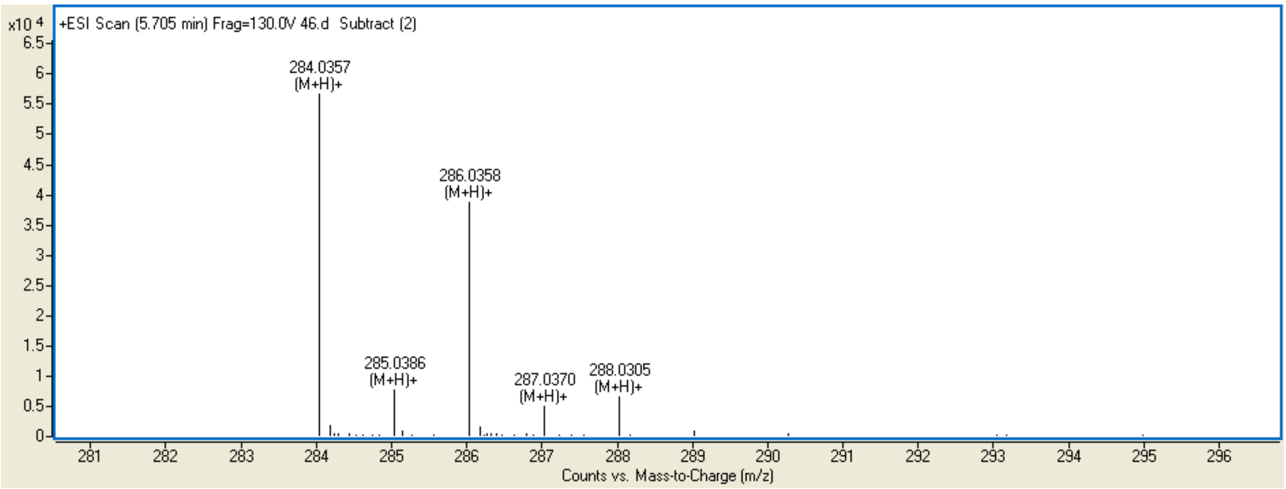

Figure S12. The HR-ESI-MS of compound **7ad**.

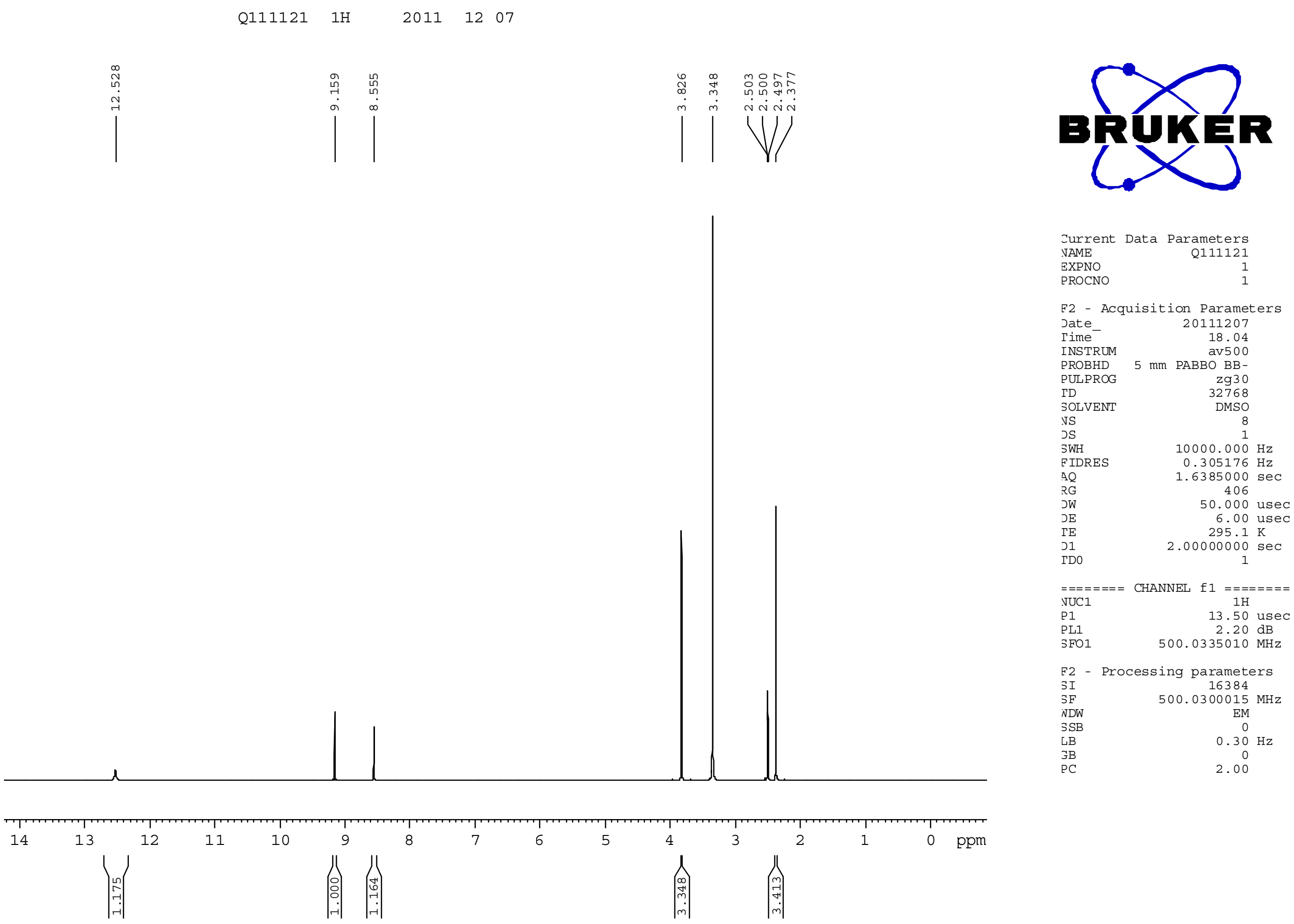

Figure S13. The ^{1}H-NMR (DMSO-d_6, 500 MHz) spectra of compound **7ae**.

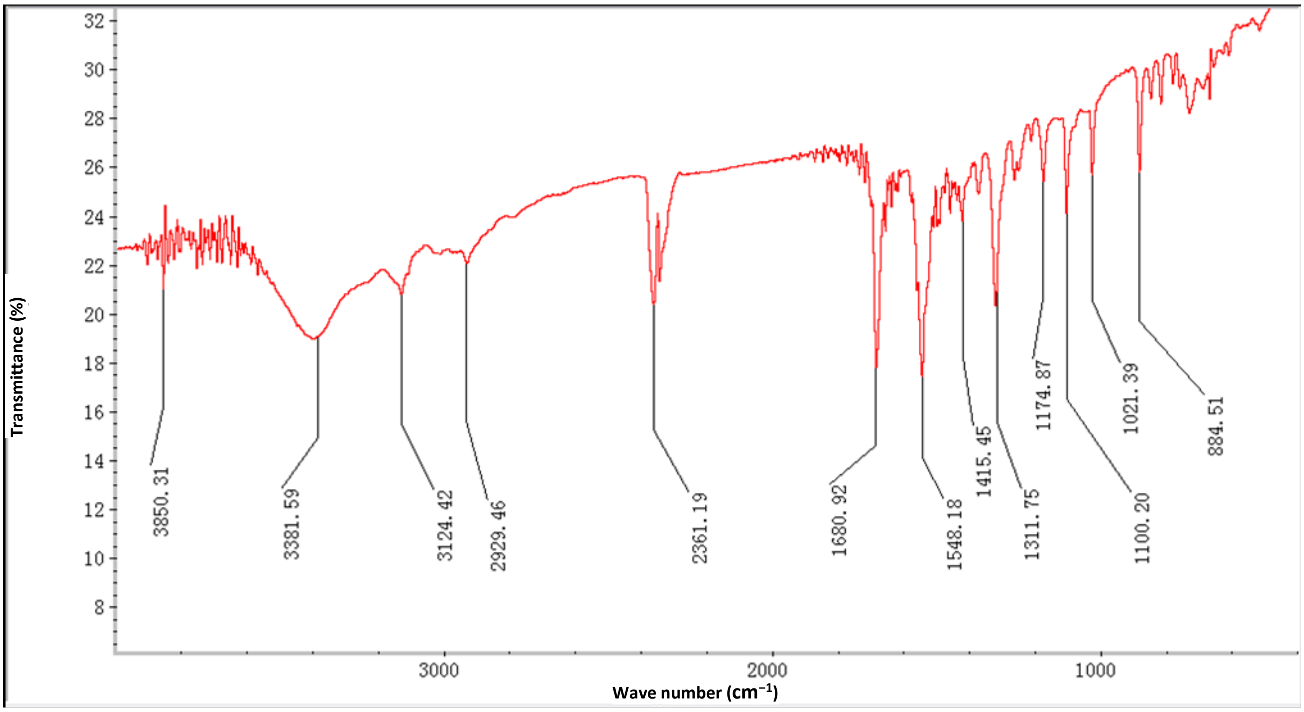

Figure S14. The IR spectra of compound **7ae**.

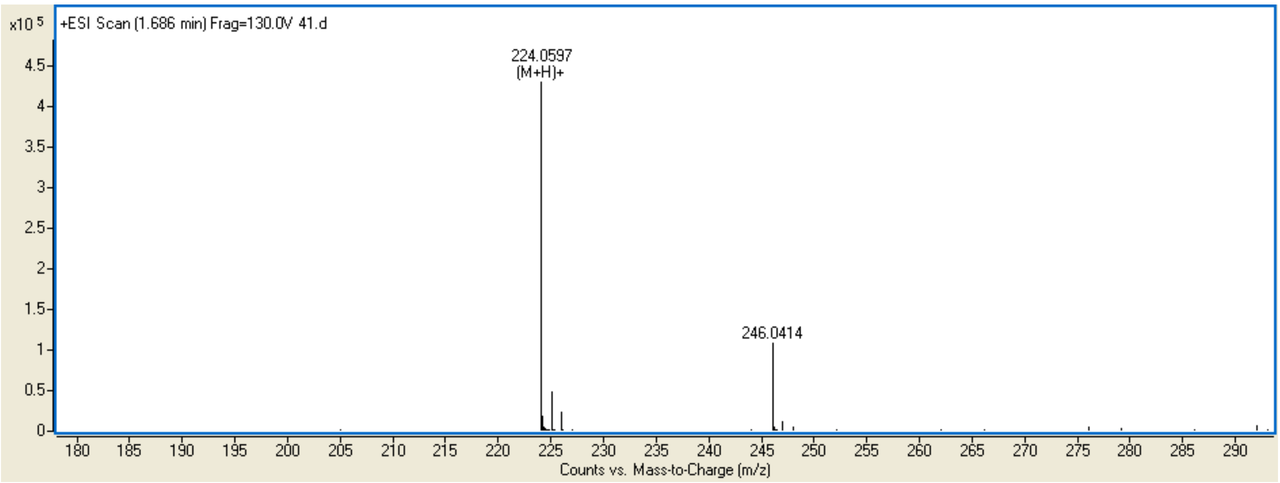

Figure S15. The HR-ESI-MS of compound **7ae**.

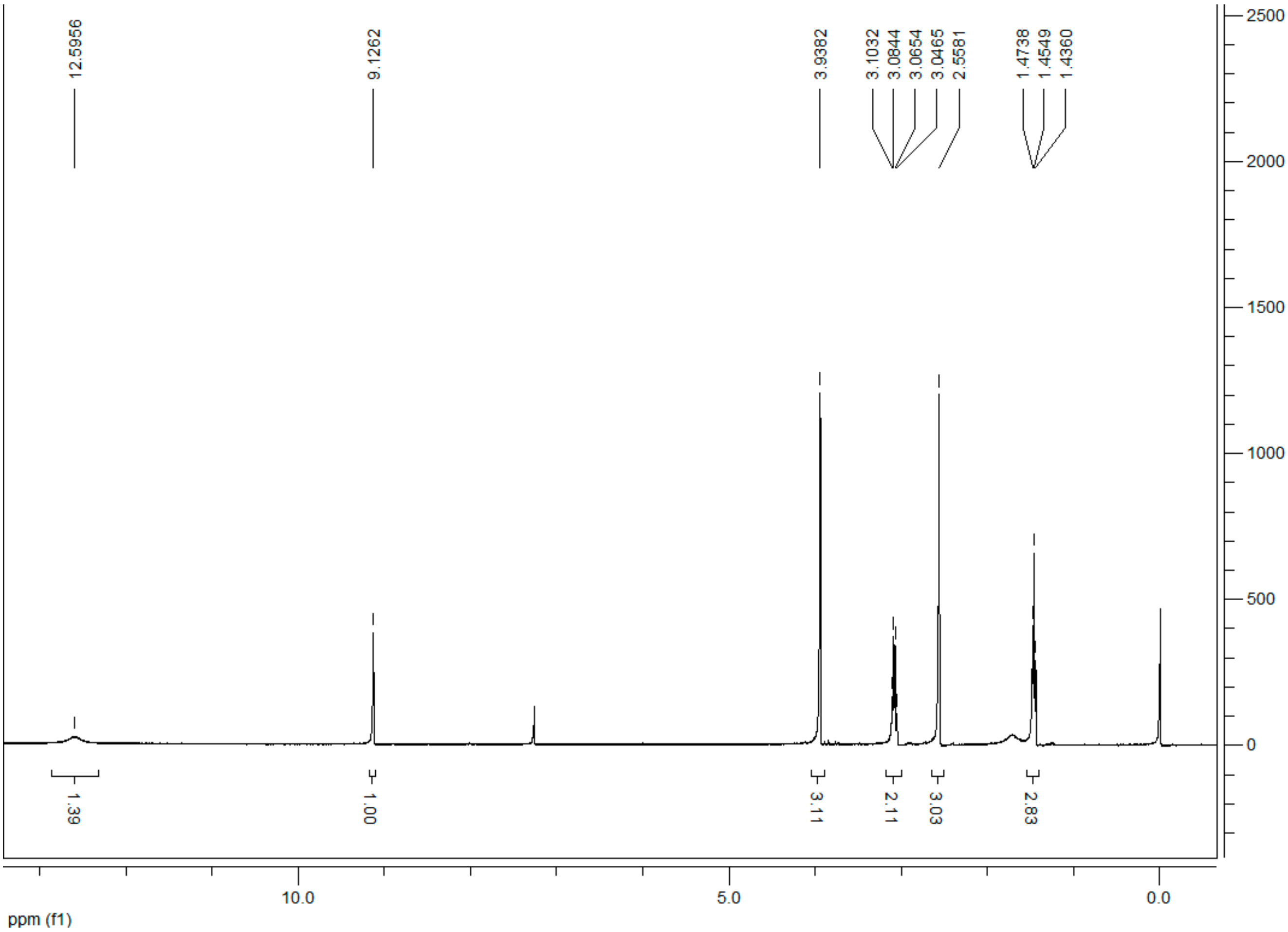

Figure S16. The ^{1}H-NMR ($CDCl_3$, 400 MHz) spectra of compound **7af**.

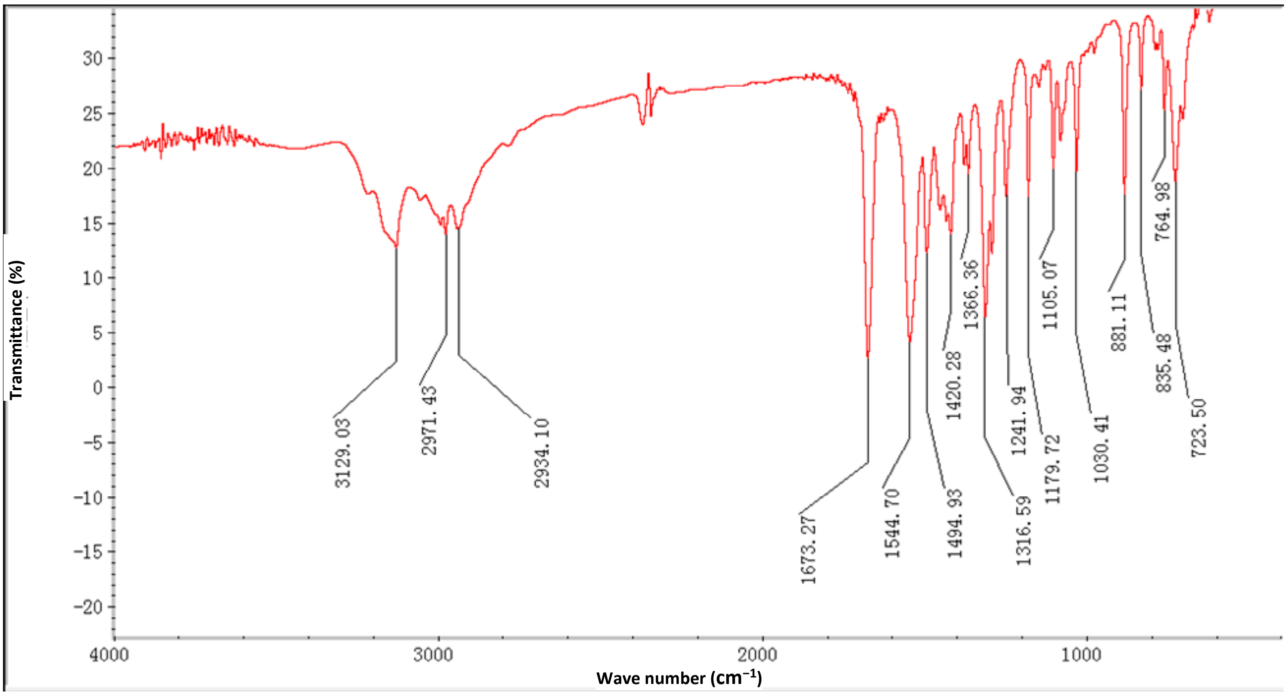

Figure S17. The IR spectra of compound **7af**.

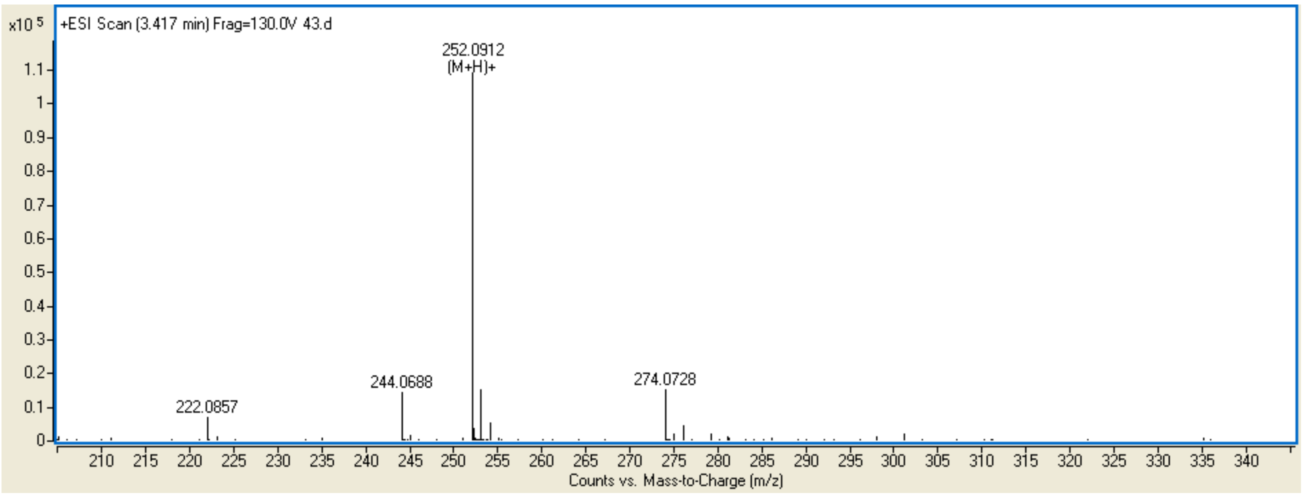

Figure S18. The HR-ESI-MS of compound **7af**.

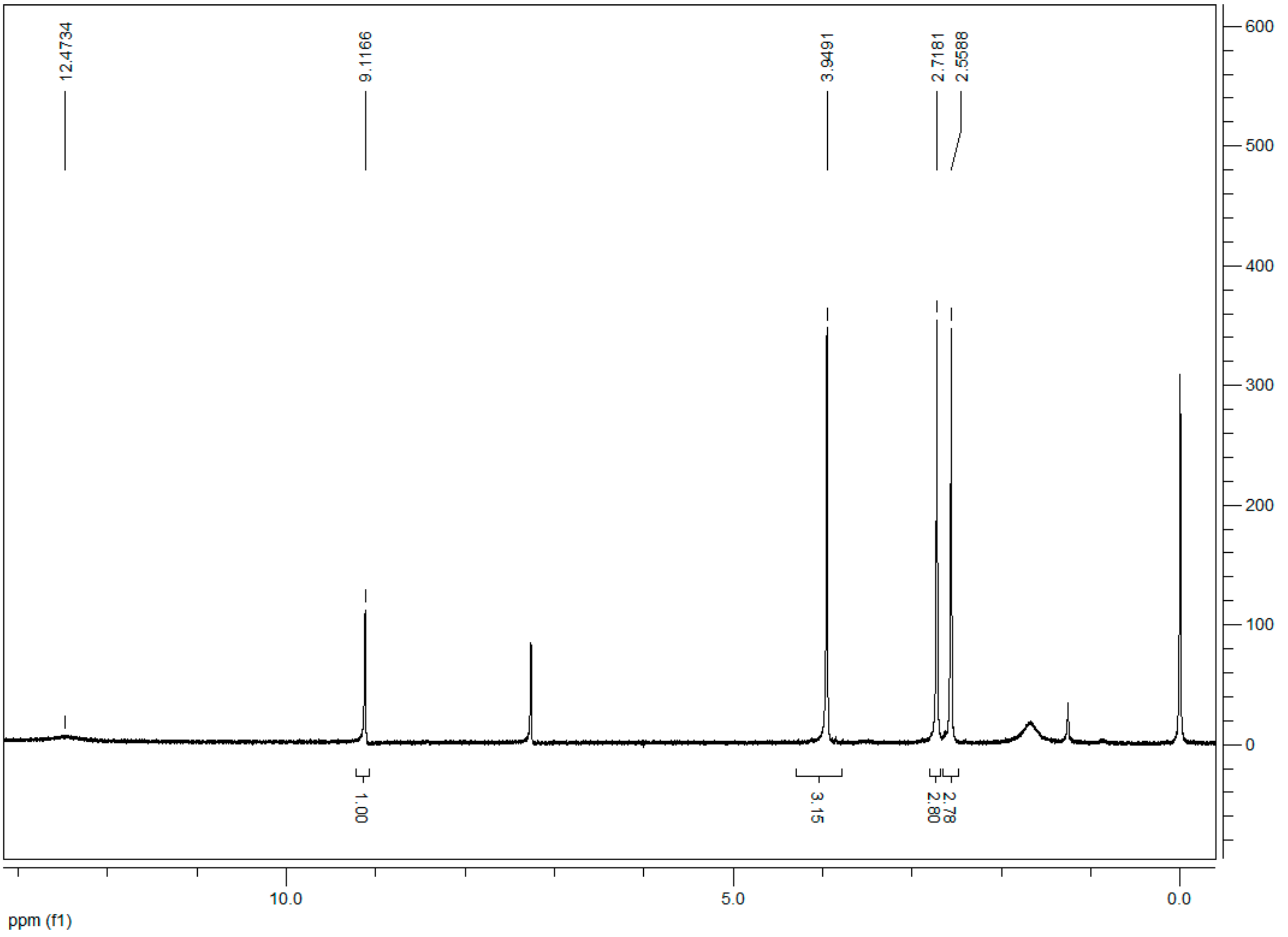

Figure S19. The ^{1}H-NMR ($CDCl_3$, 400 MHz) spectra of compound **7ag**.

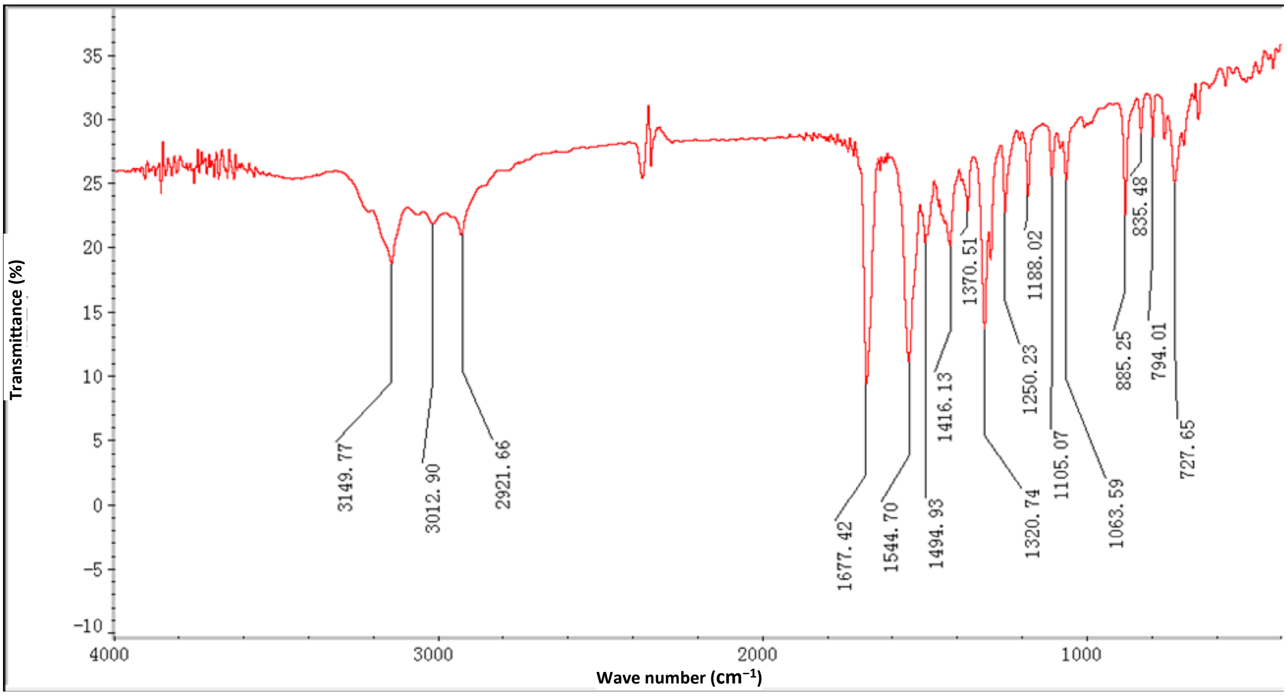

Figure S20. The IR spectra of compound **7ag**.

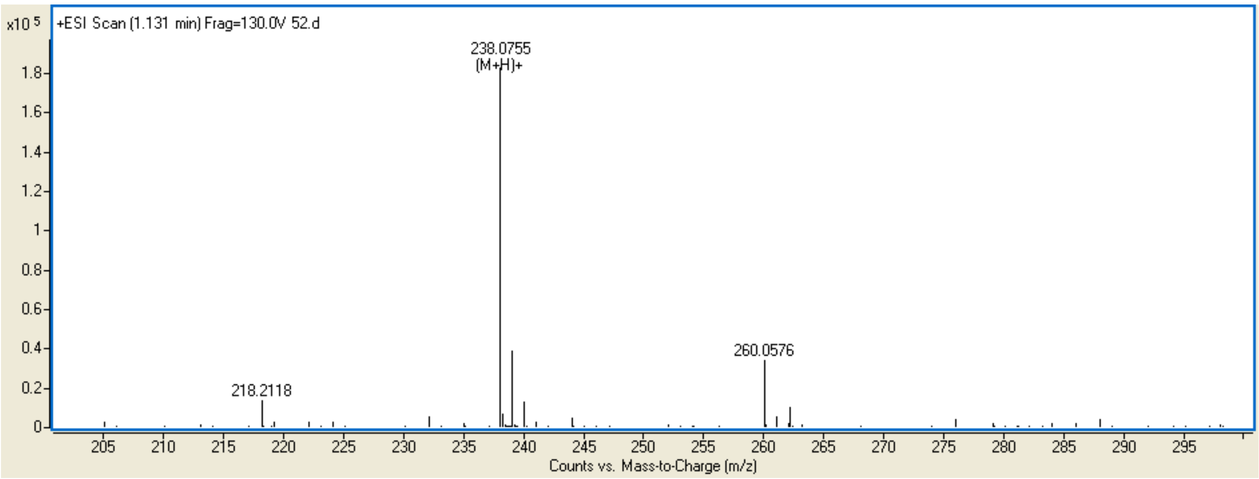

Figure S21. The HR-ESI-MS of compound **7ag**.

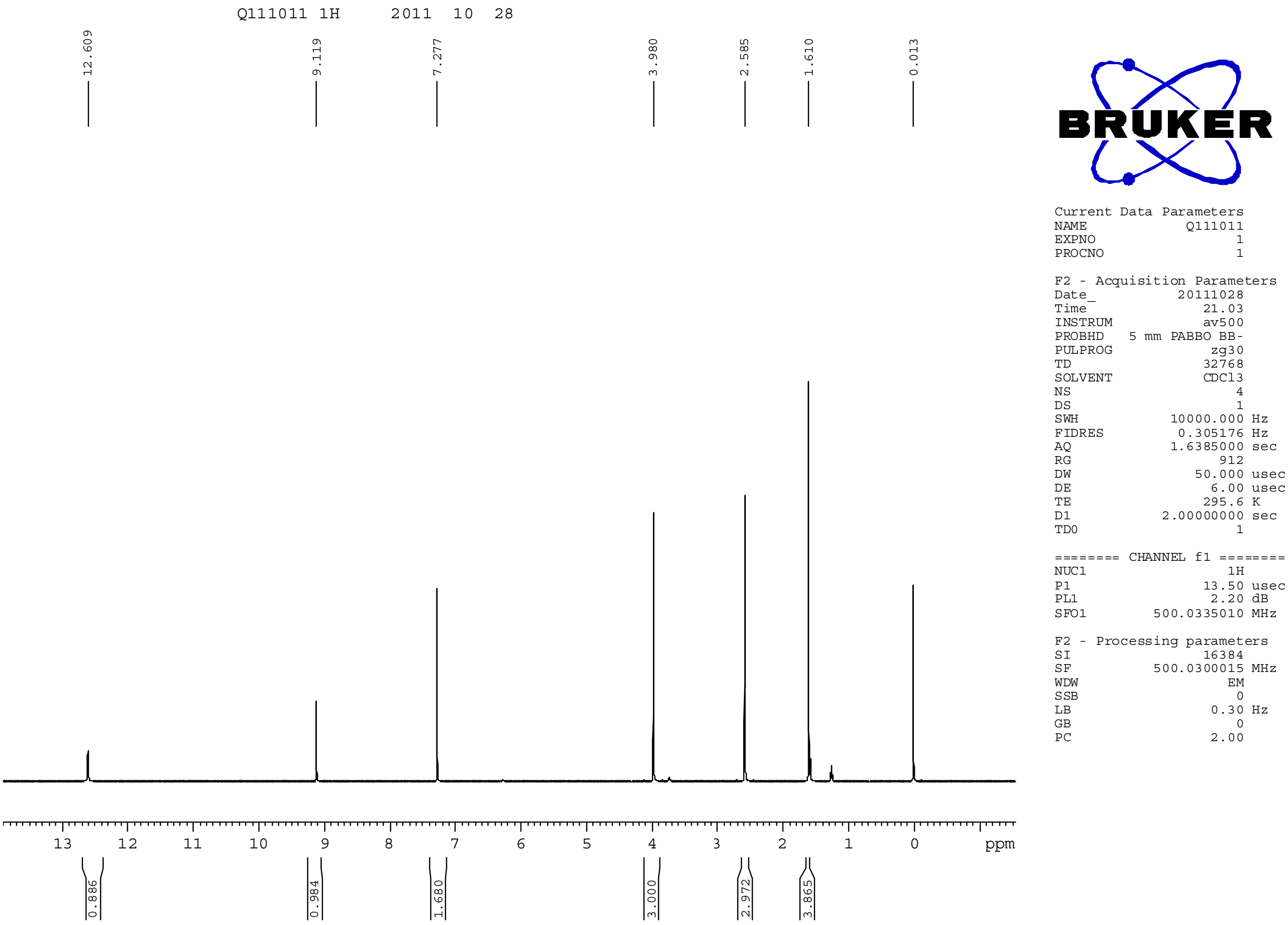

Figure S22. The ^{1}H-NMR ($CDCl_3$, 500 MHz) spectra of compound **7ah**.

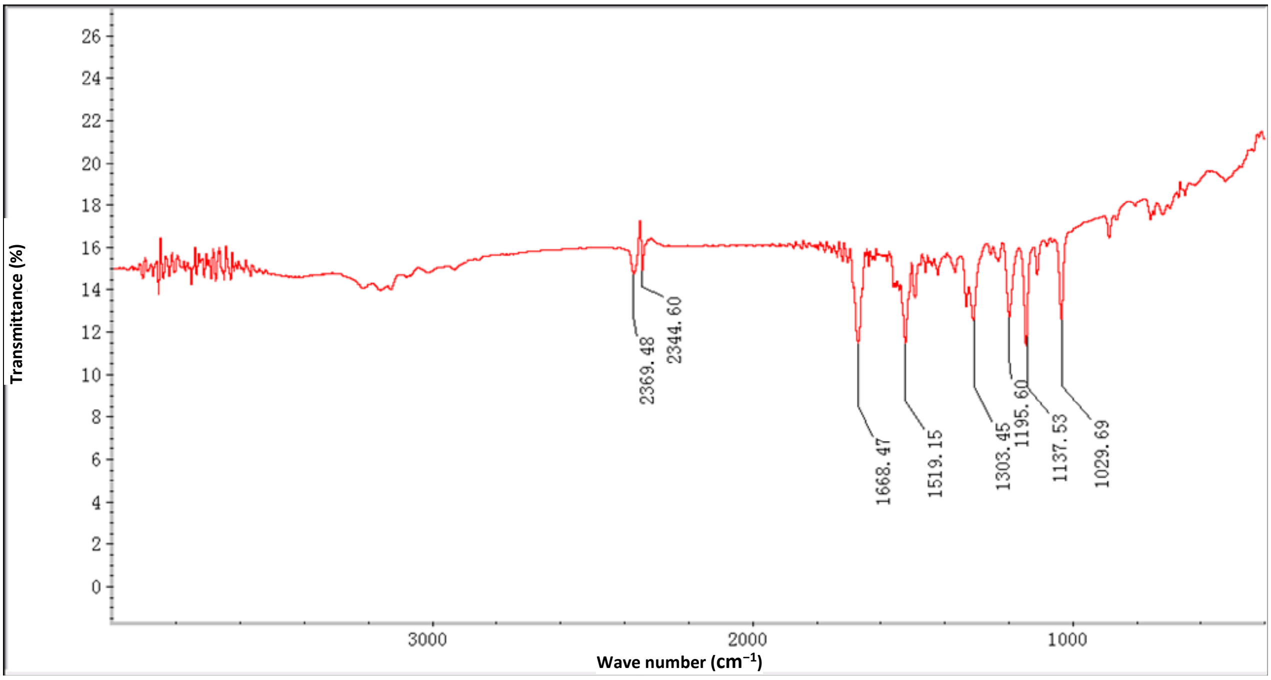

Figure S23. The IR spectra of compound **7ah**.

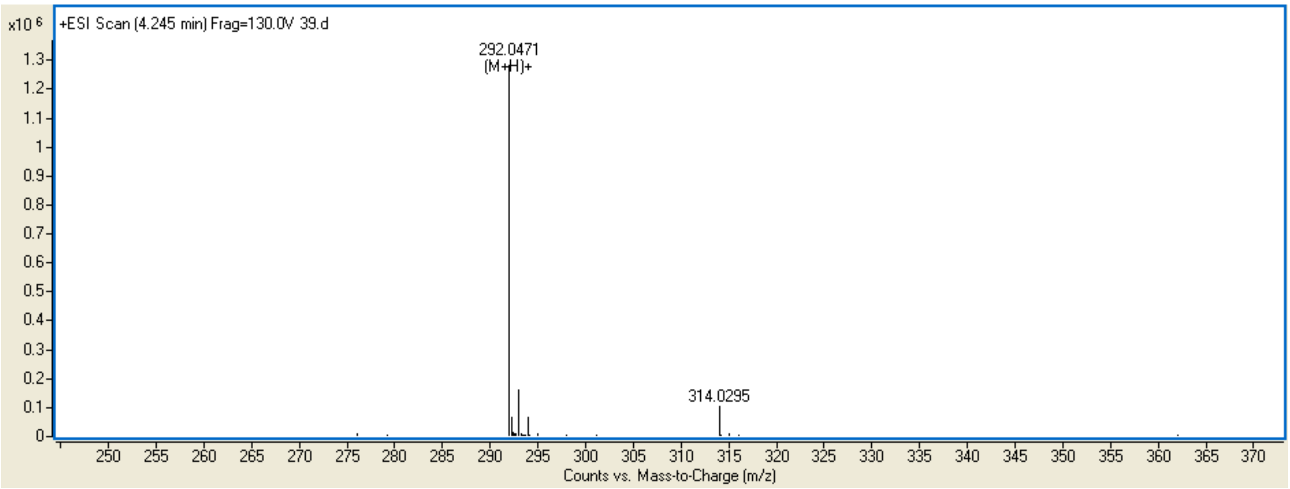

Figure S24. The HR-ESI-MS of compound **7ah**.

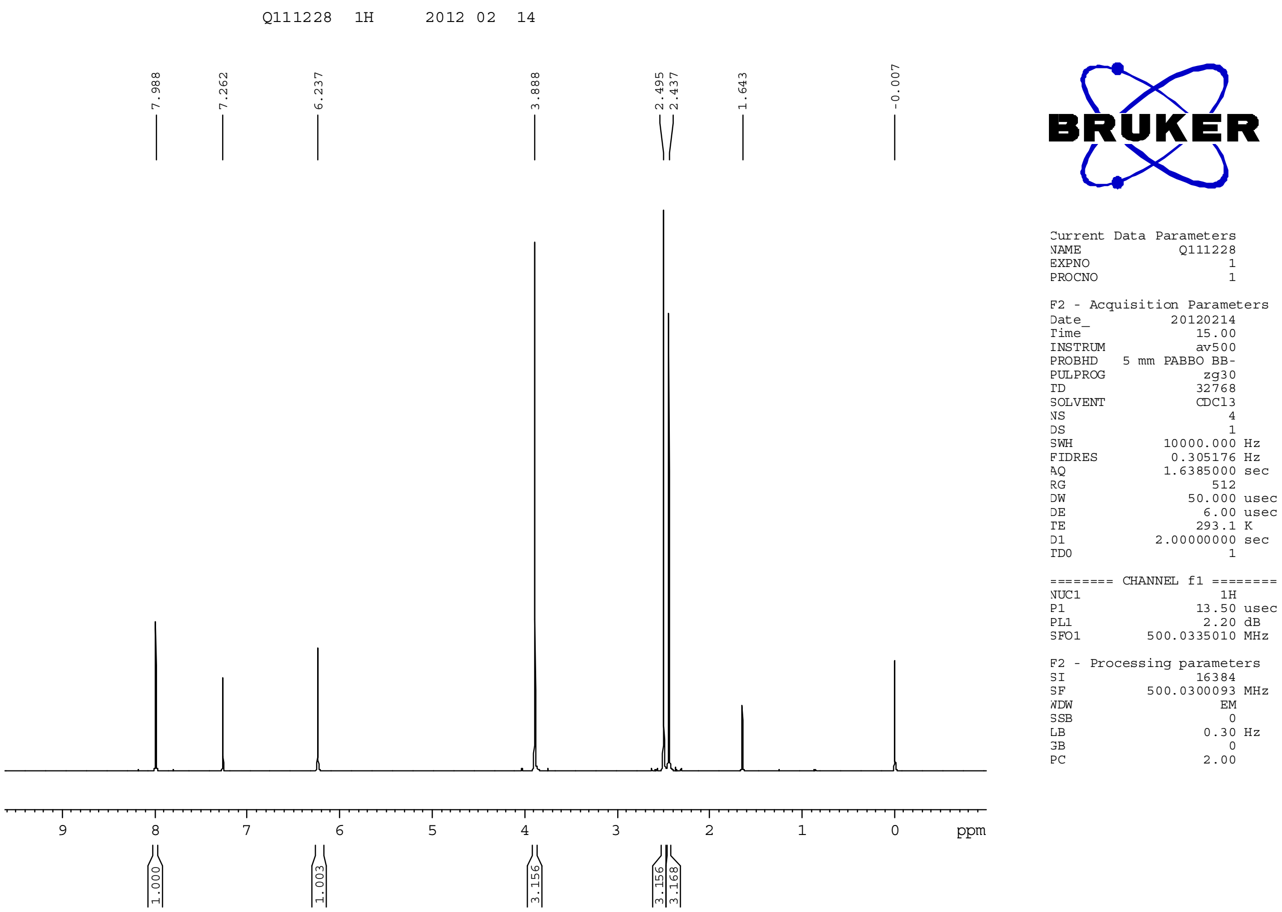

Figure S25. The ¹H-NMR ($CDCl_3$, 500 MHz) spectra of compound **7ai**.

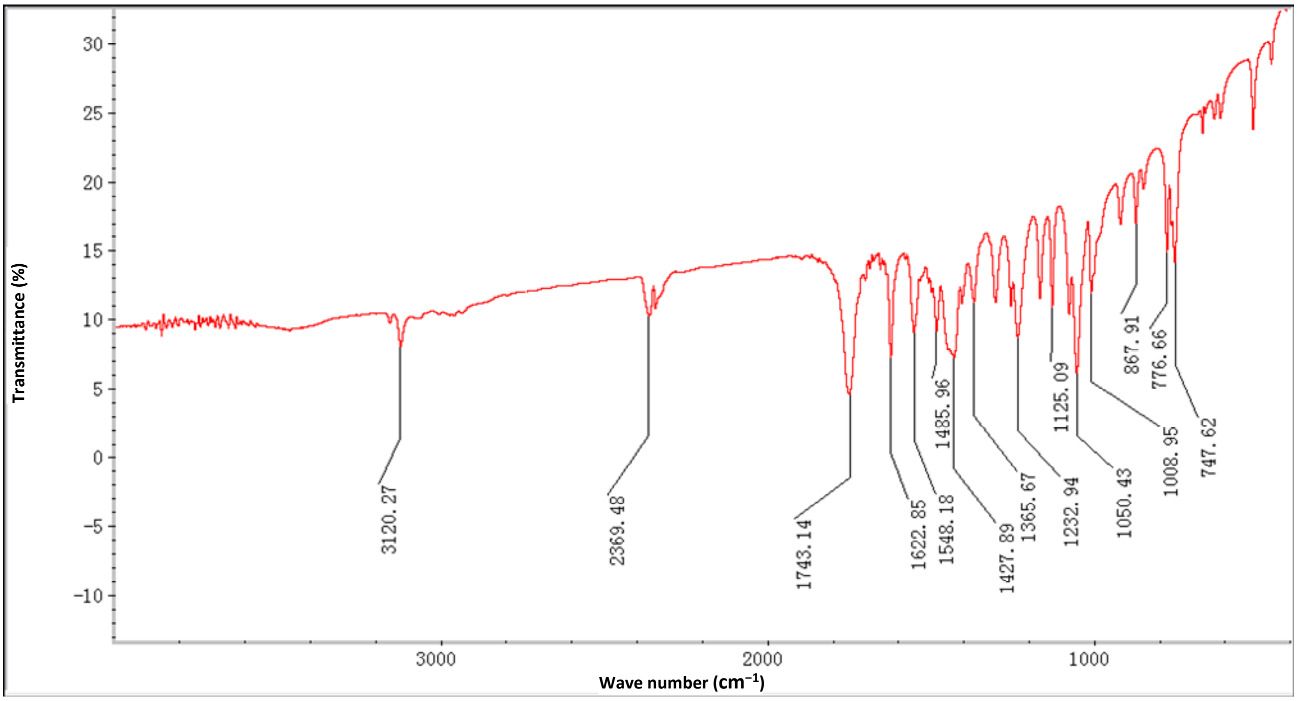

Figure S26. The IR spectra of compound **7ai**.

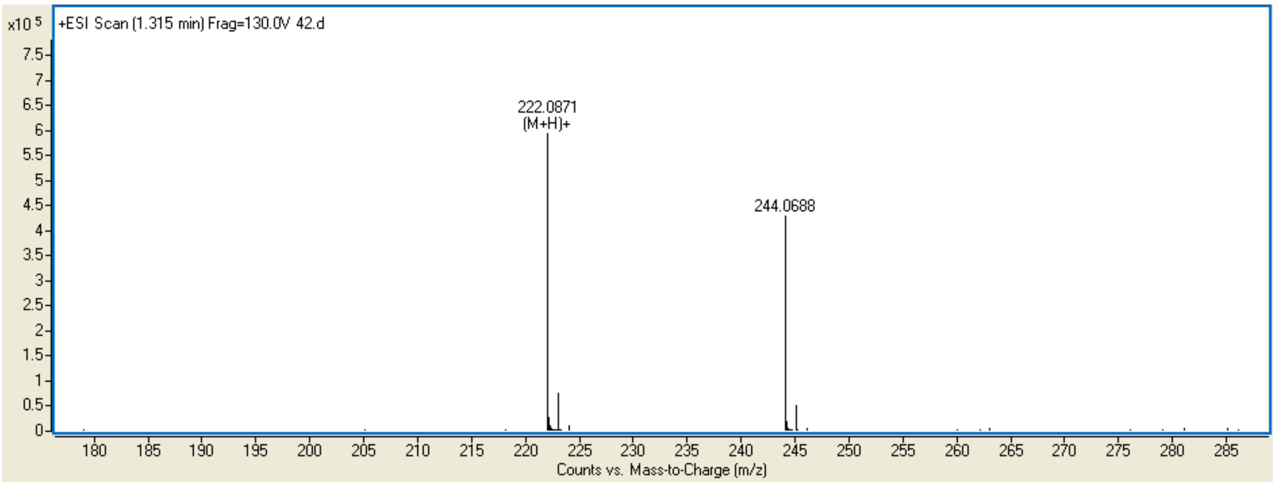

Figure S27. The HR-ESI-MS of compound **7ai**.

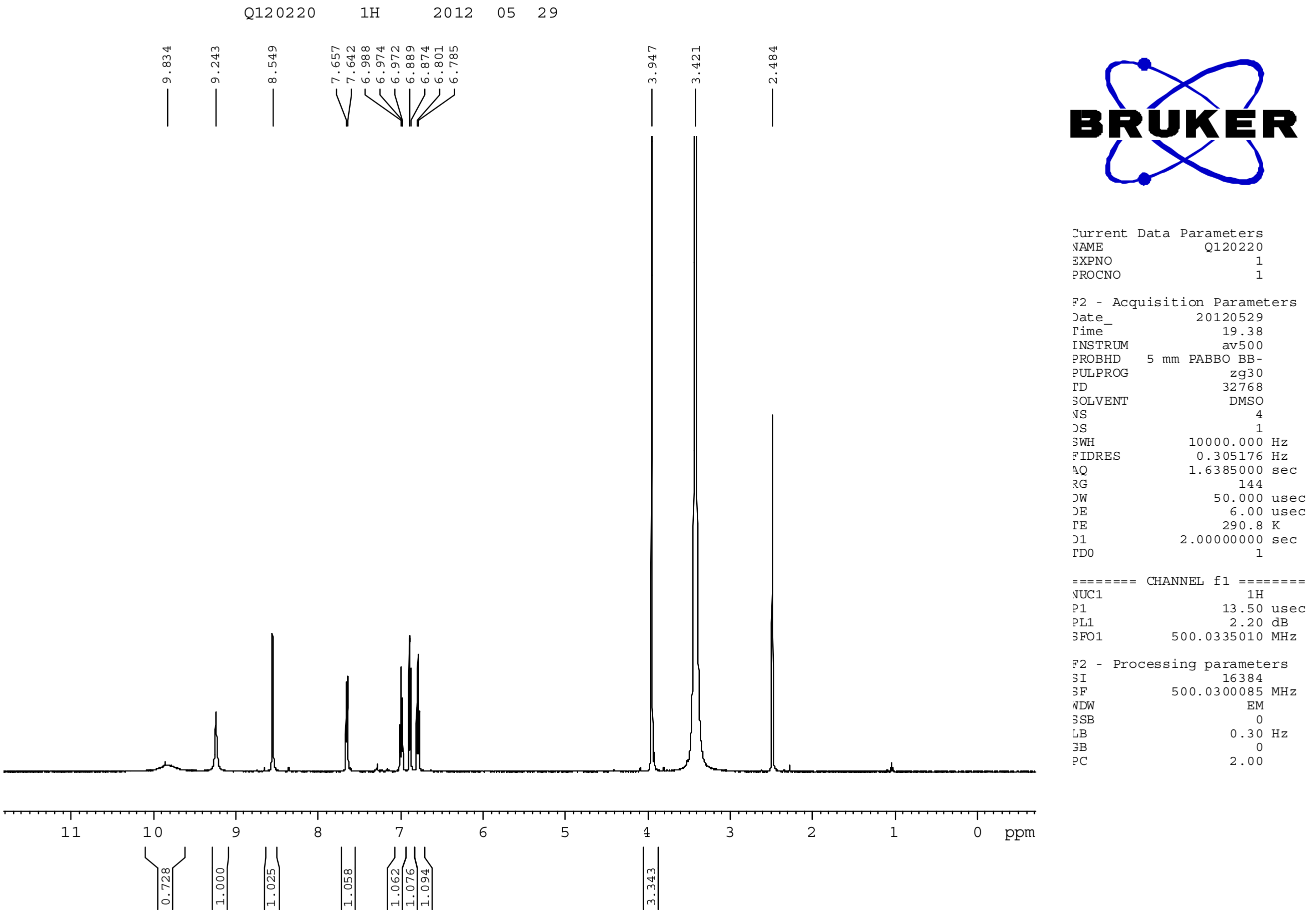

Figure S28. The ^{1}H-NMR (DMSO-d_6, 500 MHz) spectra of compound **7ba**.

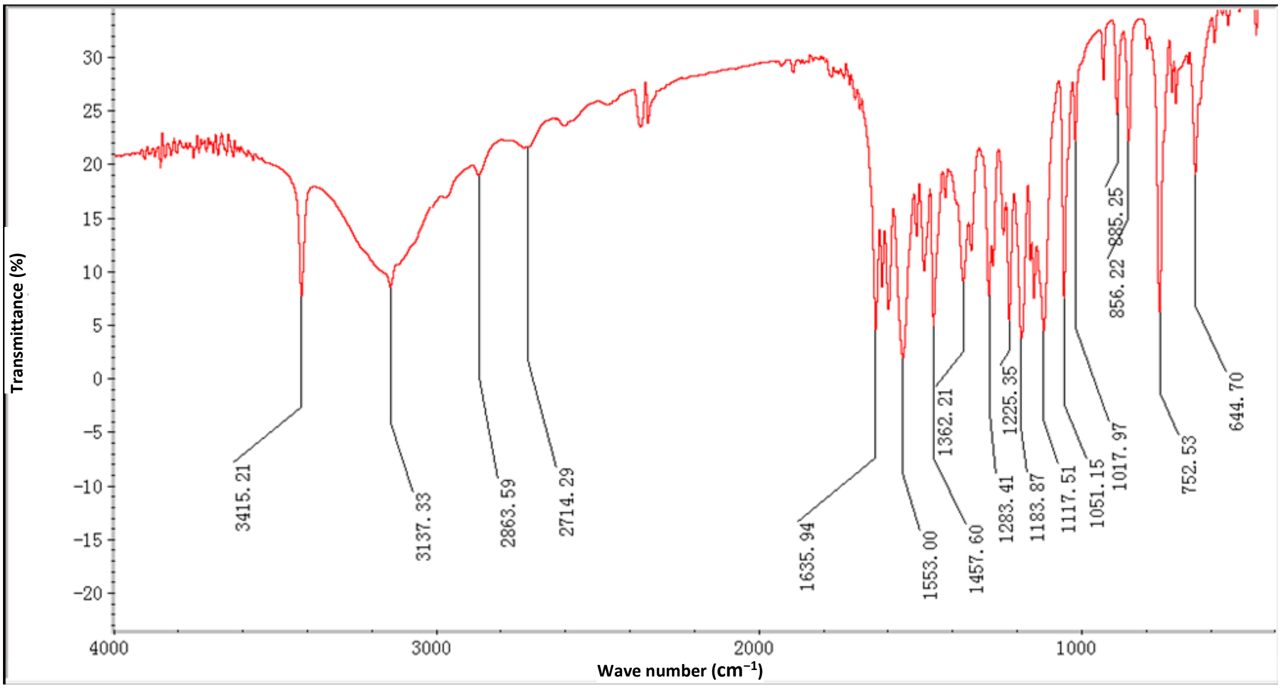

Figure S29. The IR spectra of compound **7ba**.

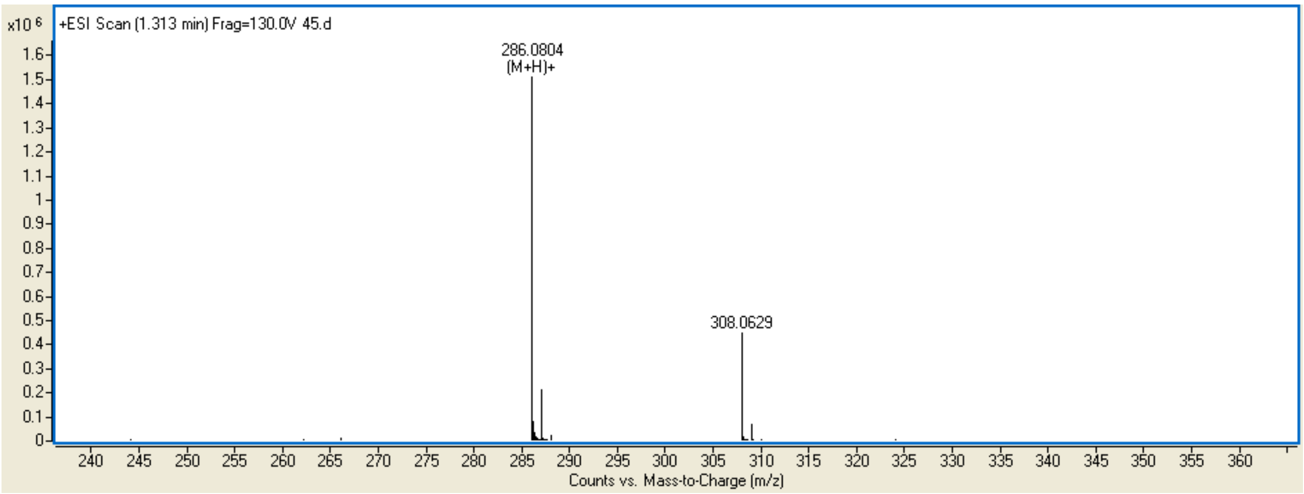

Figure S30. The HR-ESI-MS of compound **7ba**.

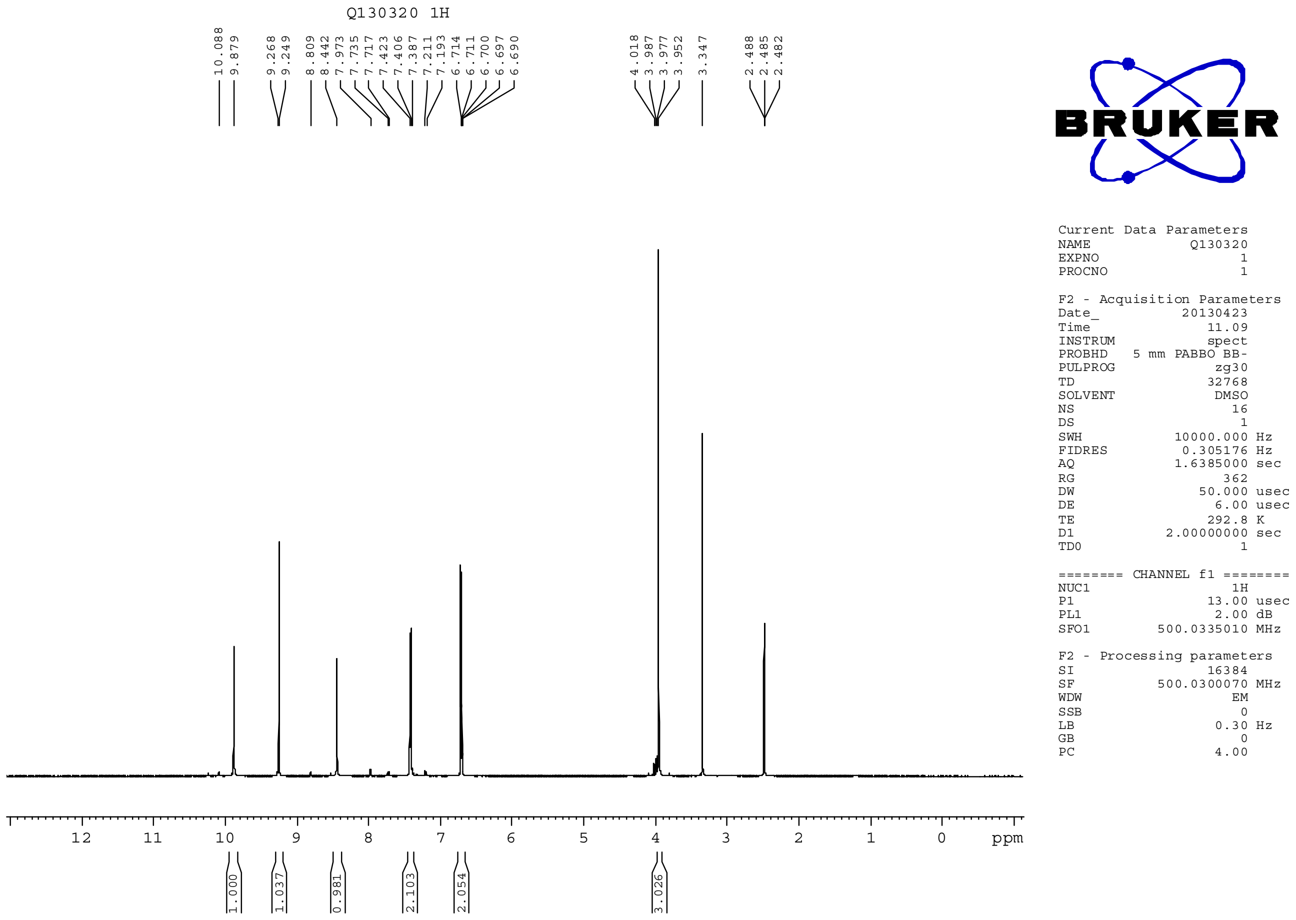

Figure S31. The ^{1}H-NMR (DMSO-d_6, 500 MHz) spectra of compound **7bb**.

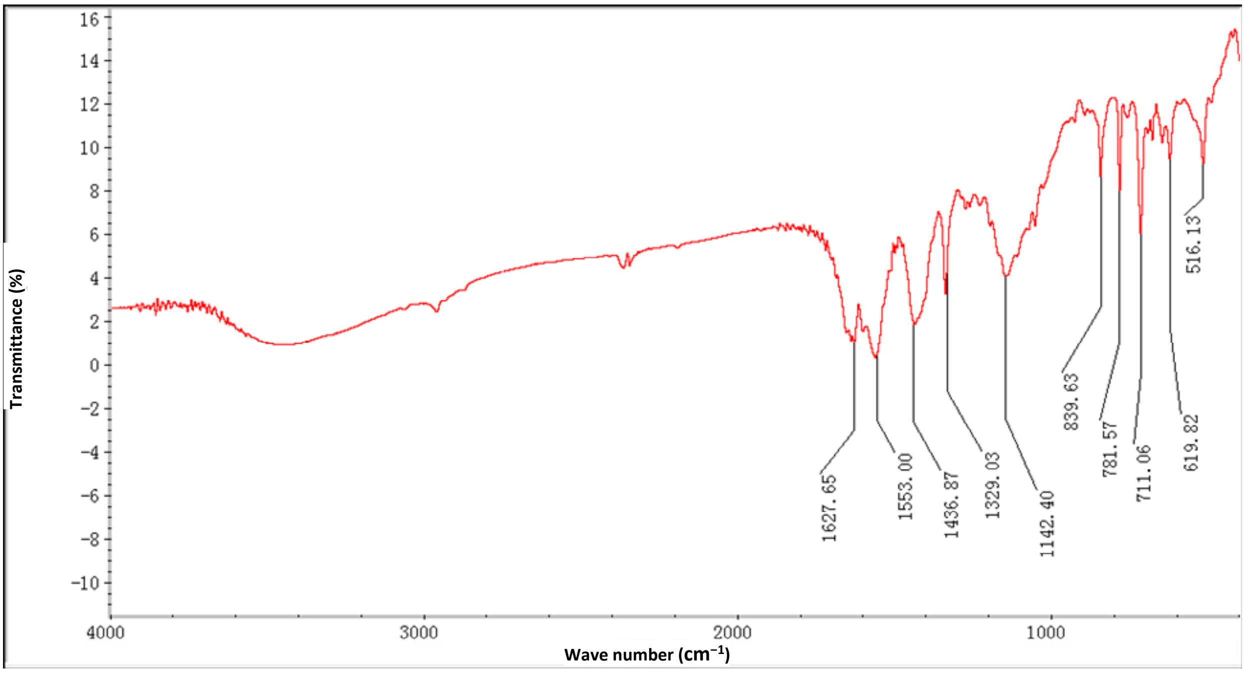

Figure S32. The IR spectra of compound **7bb**.

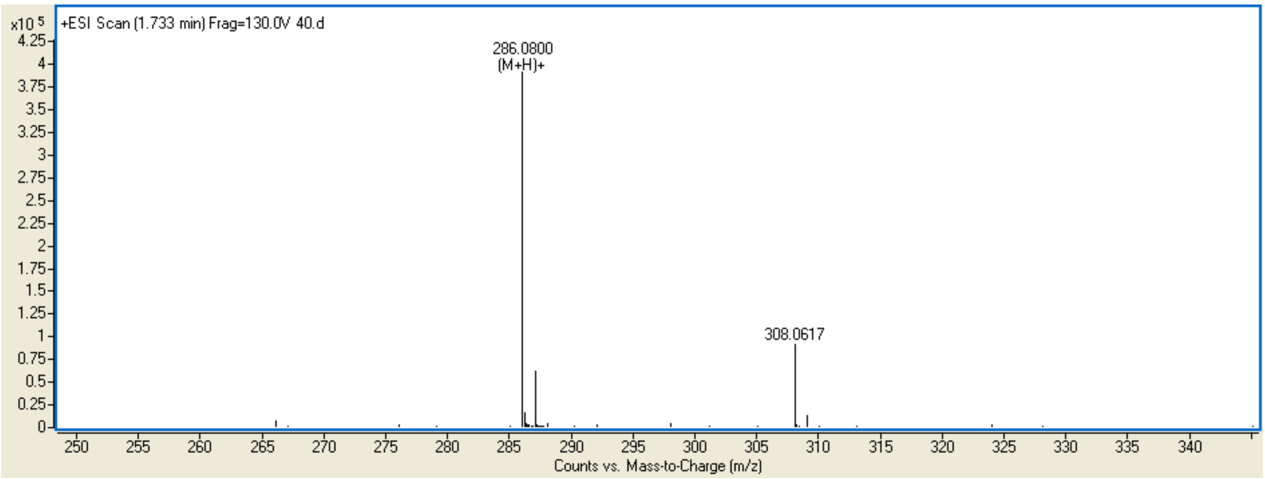

Figure S33. The HR-ESI-MS of compound **7bb**.

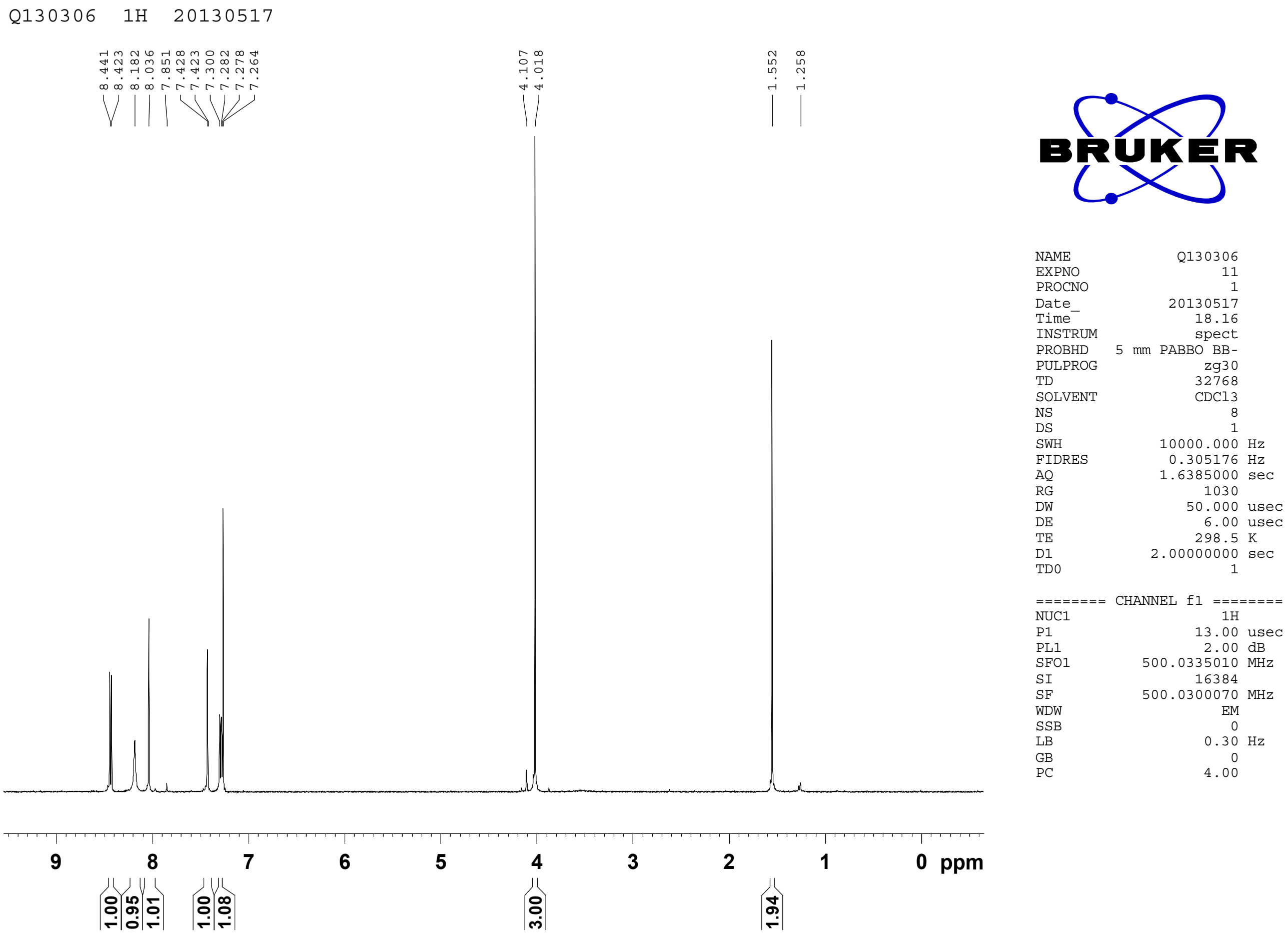

Figure S34. The ^{1}H-NMR ($CDCl_3$, 400 MHz) spectra of compound **7bc**.

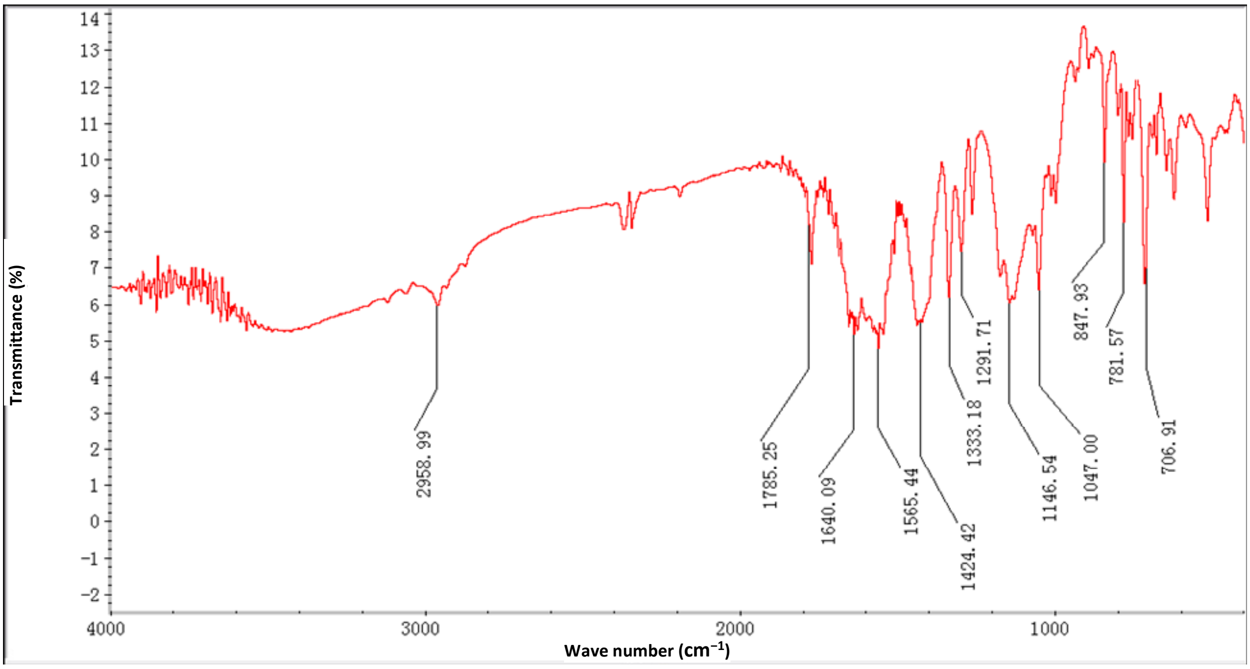

Figure S35. The IR spectra of compound **7bc**.

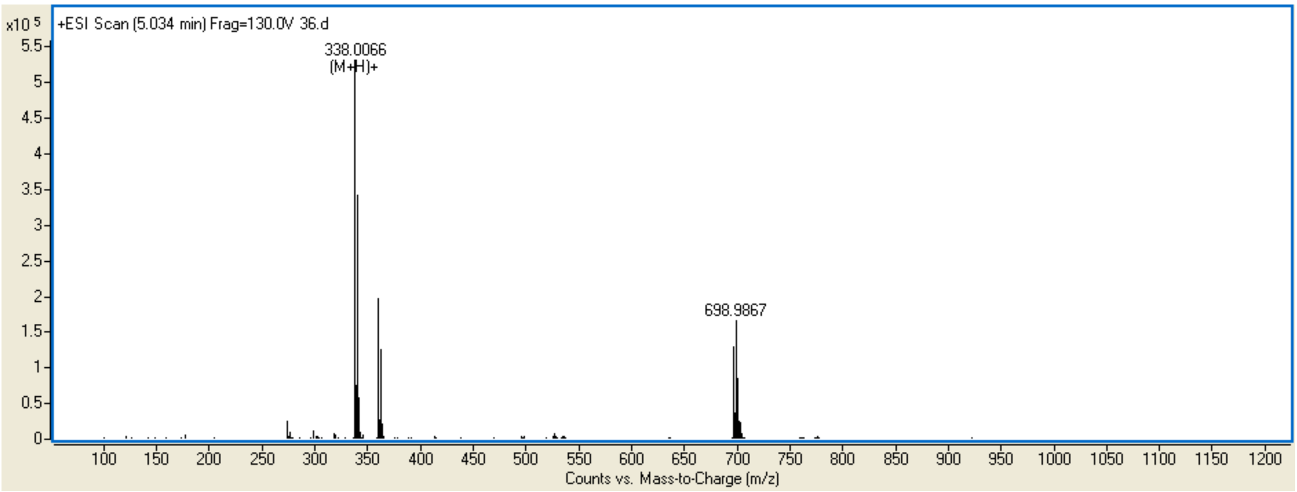

Figure S36. The HR-ESI-MS of compound **7bc**.

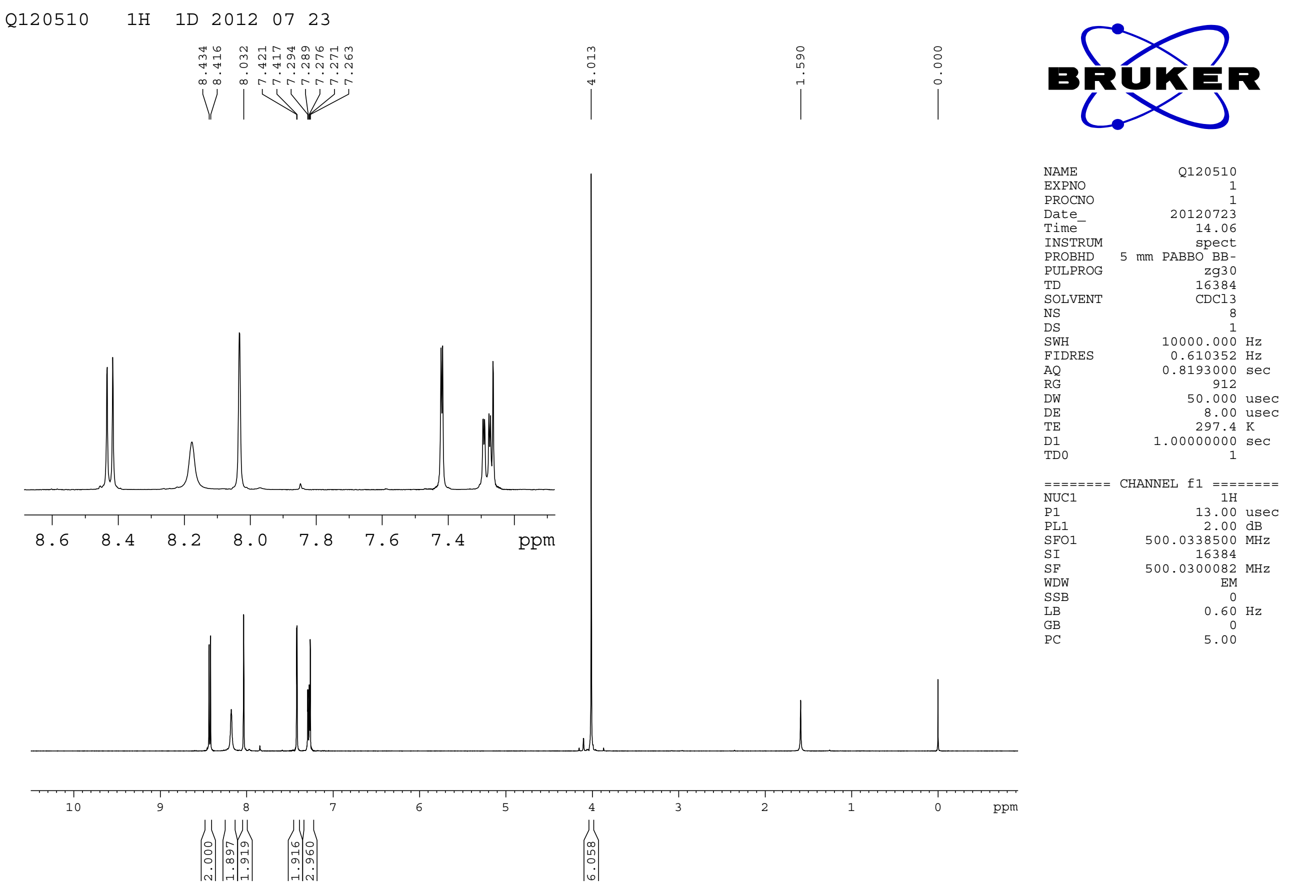

Figure S37. The ^{1}H-NMR ($CDCl_3$, 500 MHz) spectra of compound **7bd**.

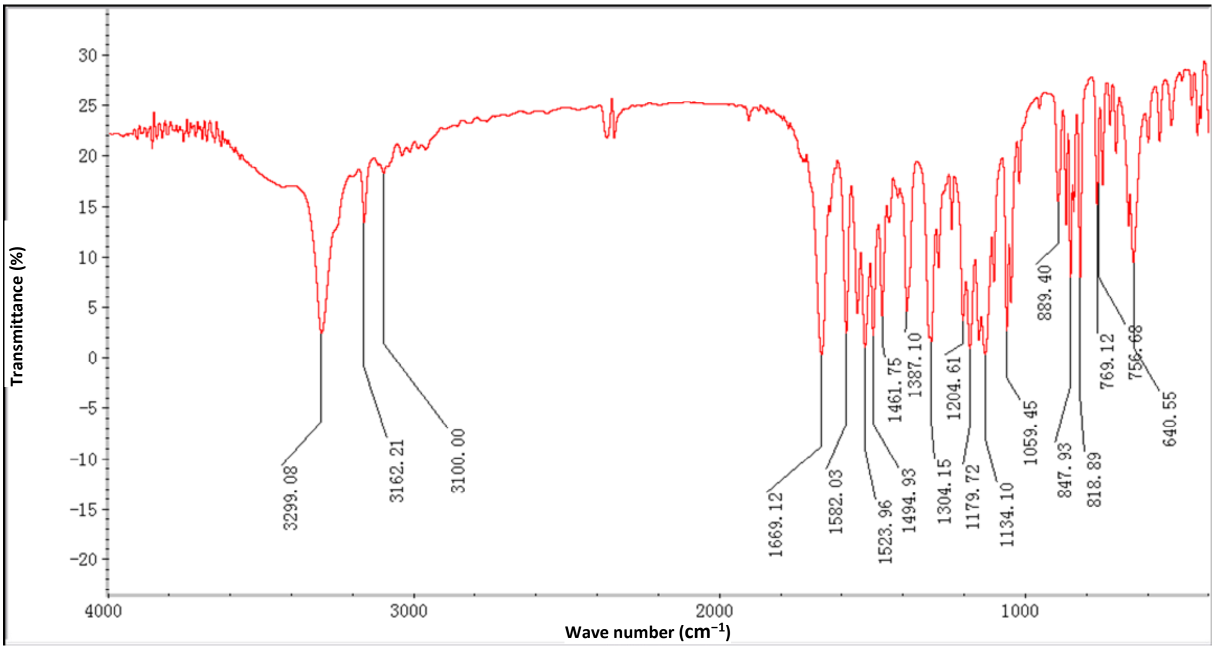

Figure S38. The IR spectra of compound **7bd**.

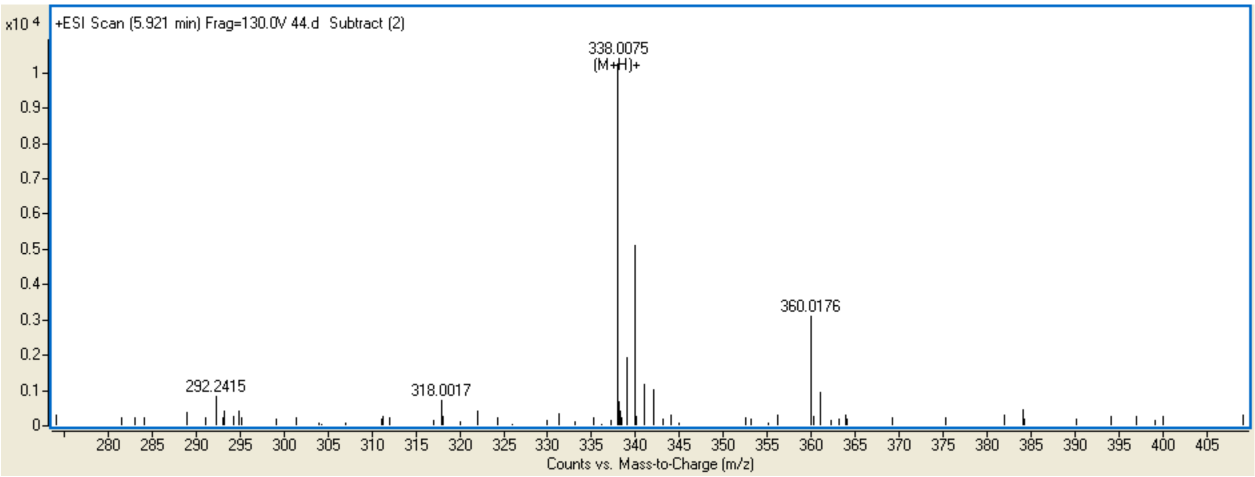

Figure S39. The HR-ESI-MS of compound **7bd**.

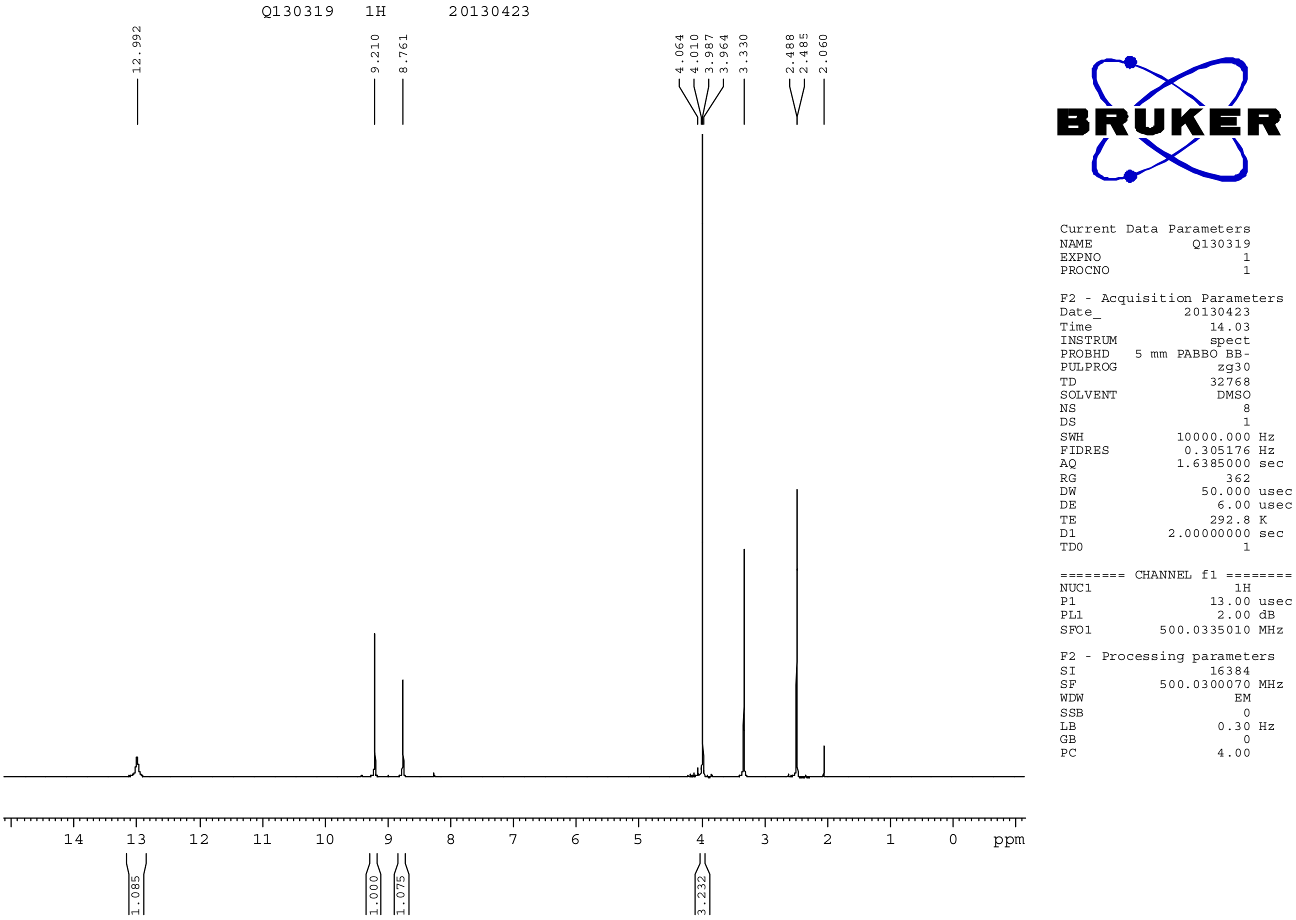

Figure S40. The ^{1}H-NMR (DMSO-d_6, 500 MHz) spectra of compound **7be**.

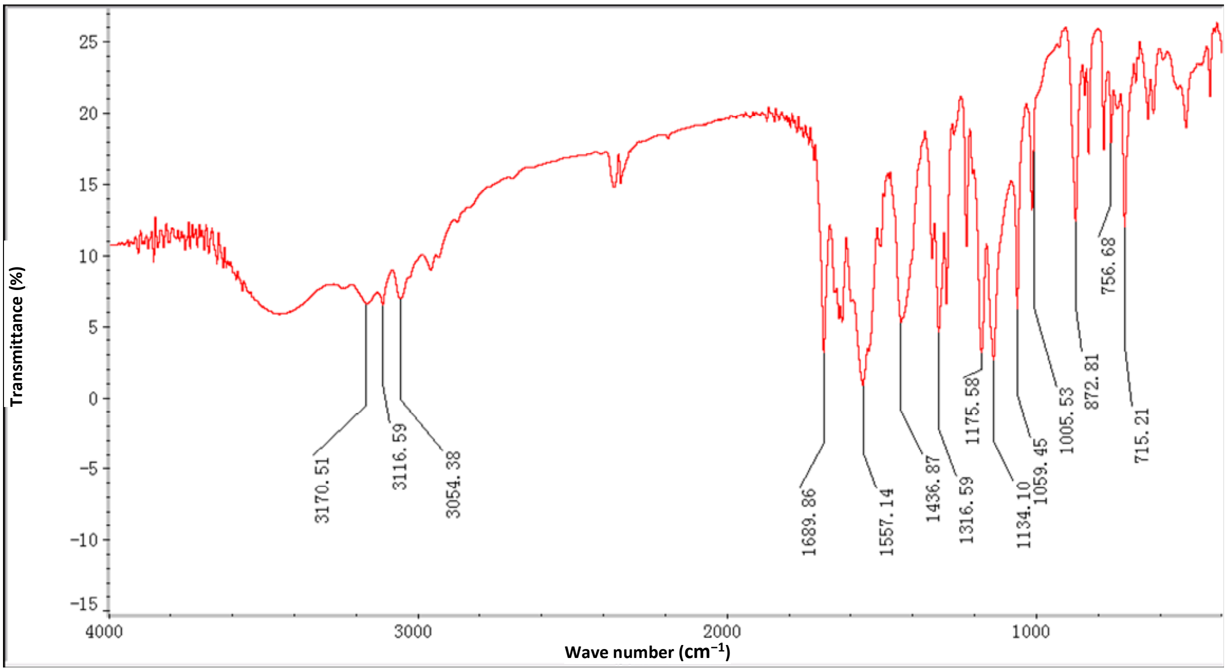

Figure S41. The IR spectra of compound **7bd**.

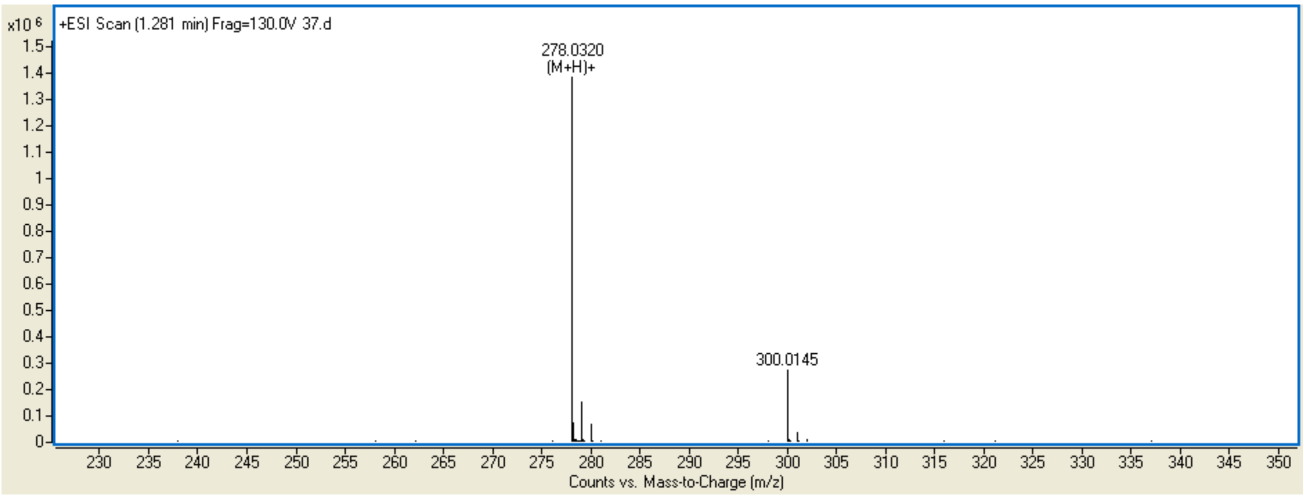

Figure S42. The HR-ESI-MS of compound **7be**.

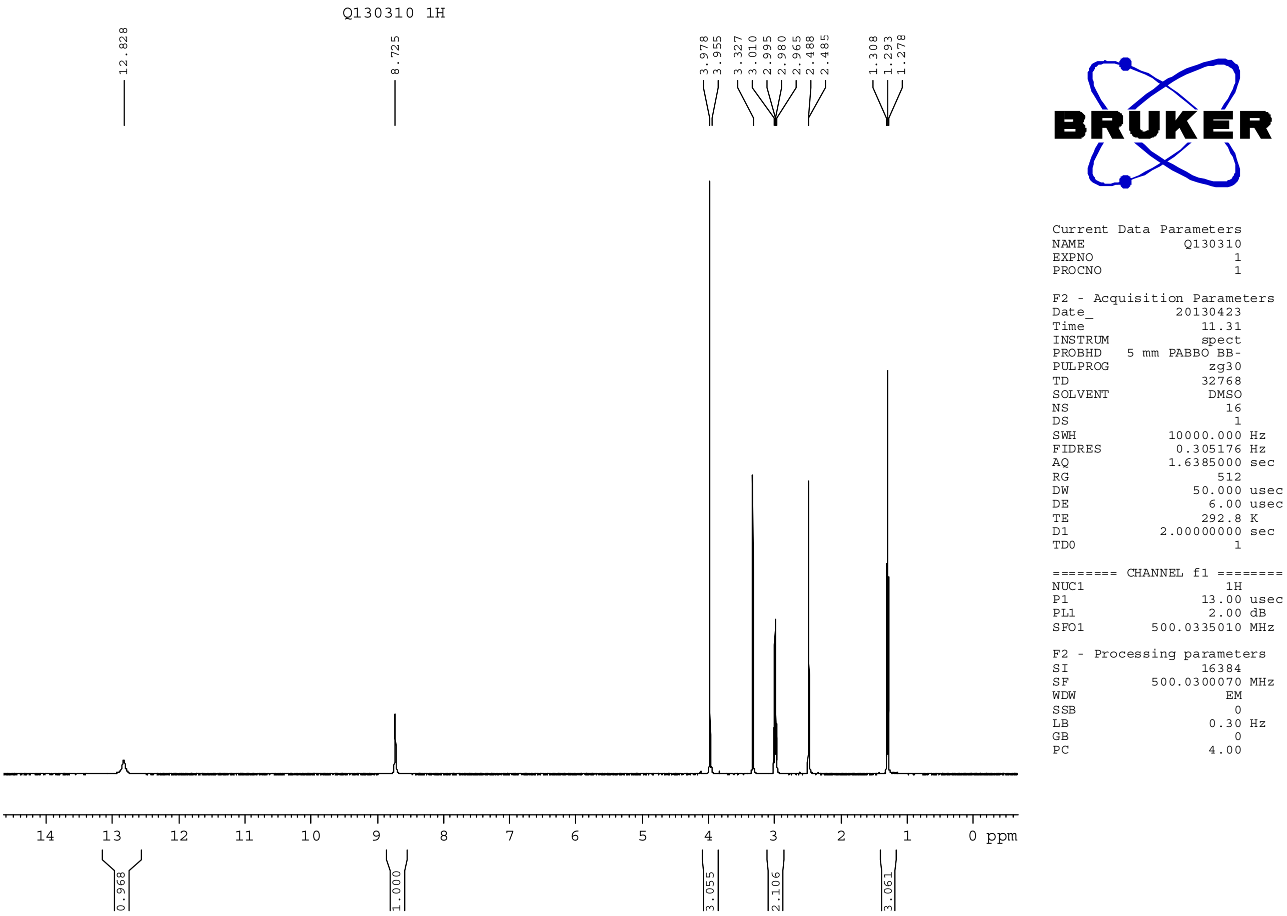

Figure S43. The ^{1}H-NMR (DMSO-d_6, 500 MHz) spectra of compound **7bf**.

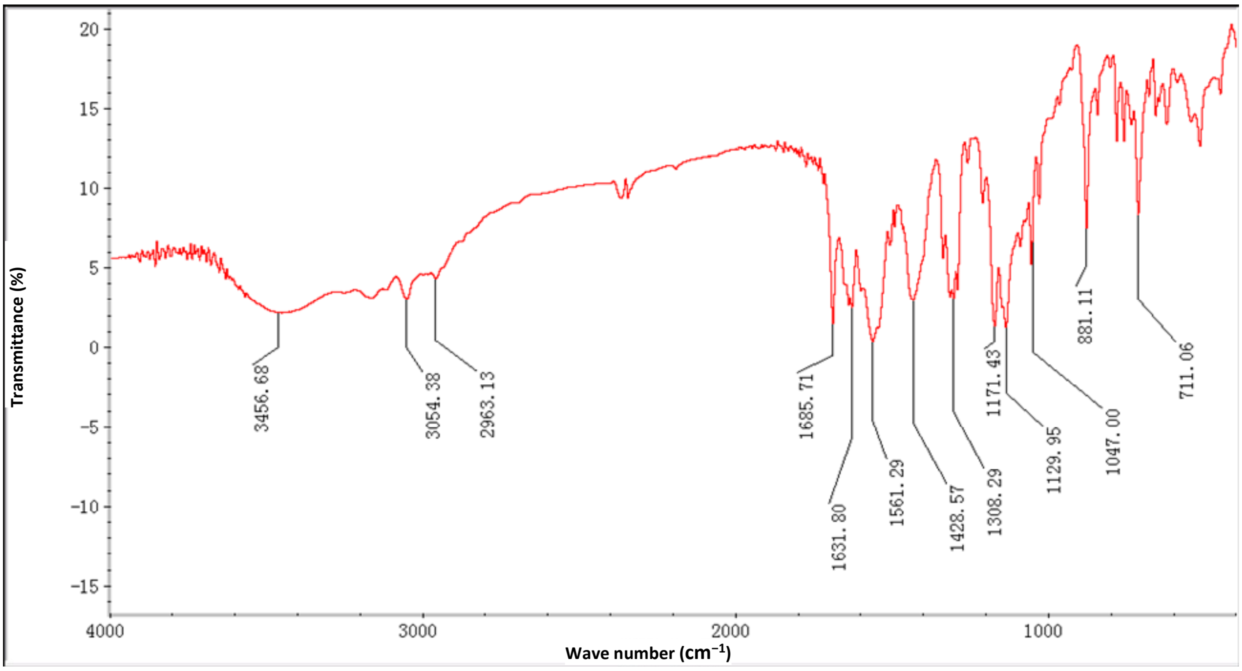

Figure S44. The IR spectra of compound **7bf**.

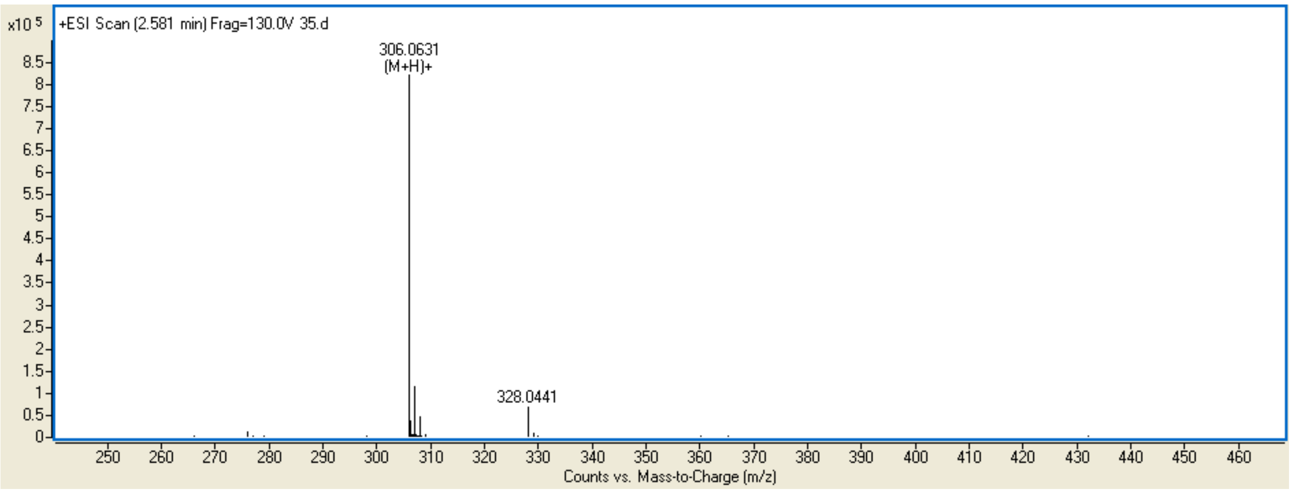

Figure S45. The HR-ESI-MS of compound **7bf**.

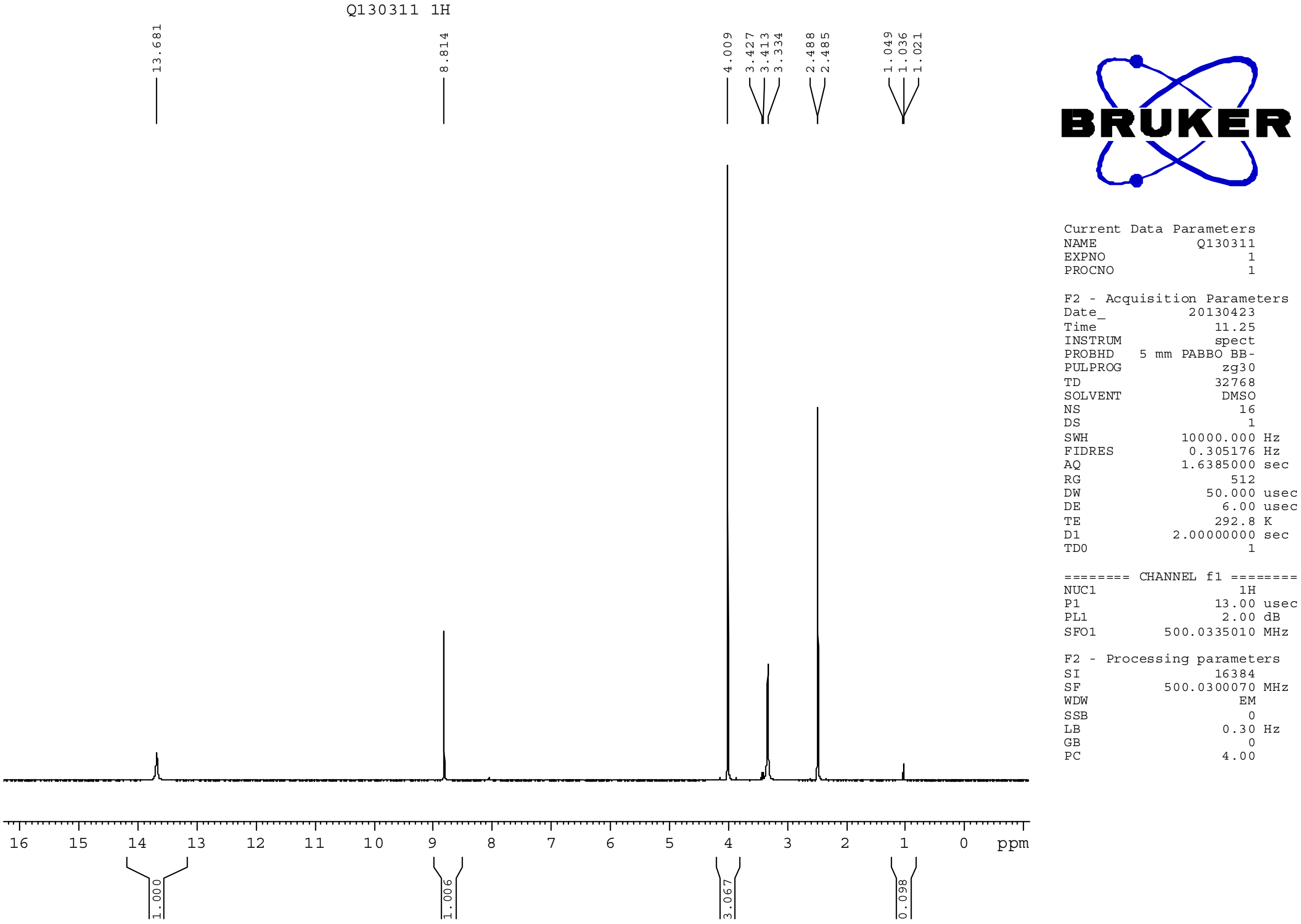

Figure S46. The ^{1}H-NMR (DMSO-d_6, 500 MHz) spectra of compound **7bg**.

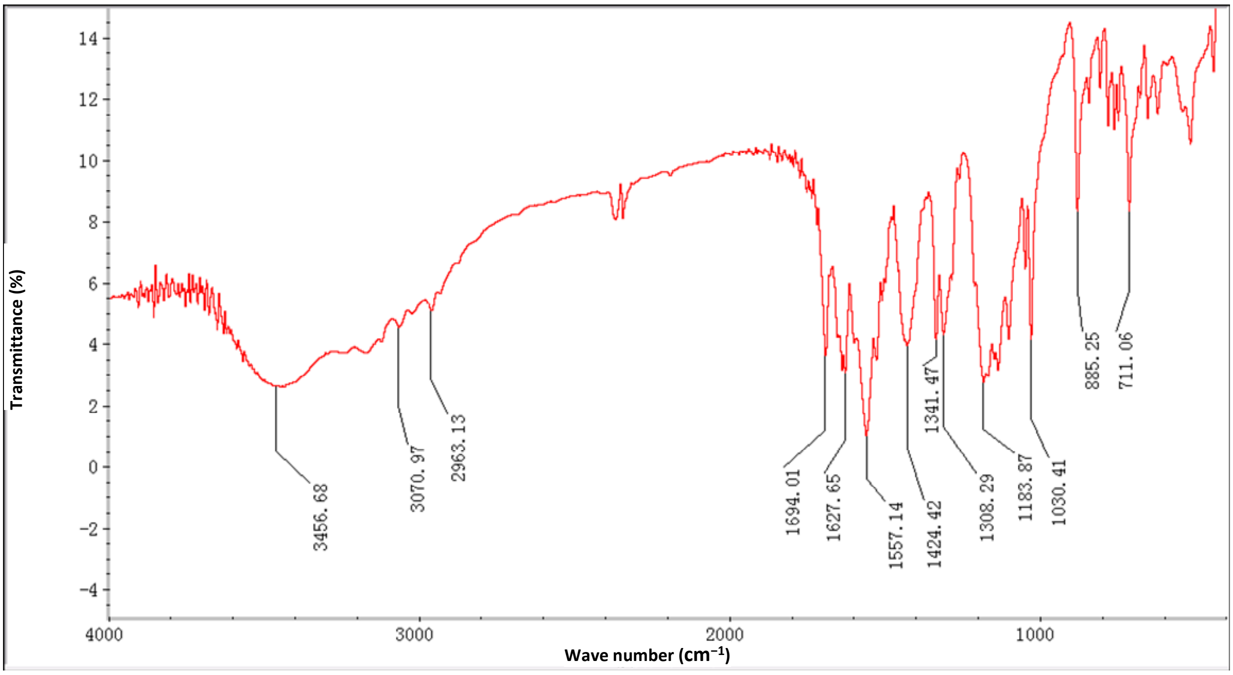

Figure S47. The IR spectra of compound **7bg**.

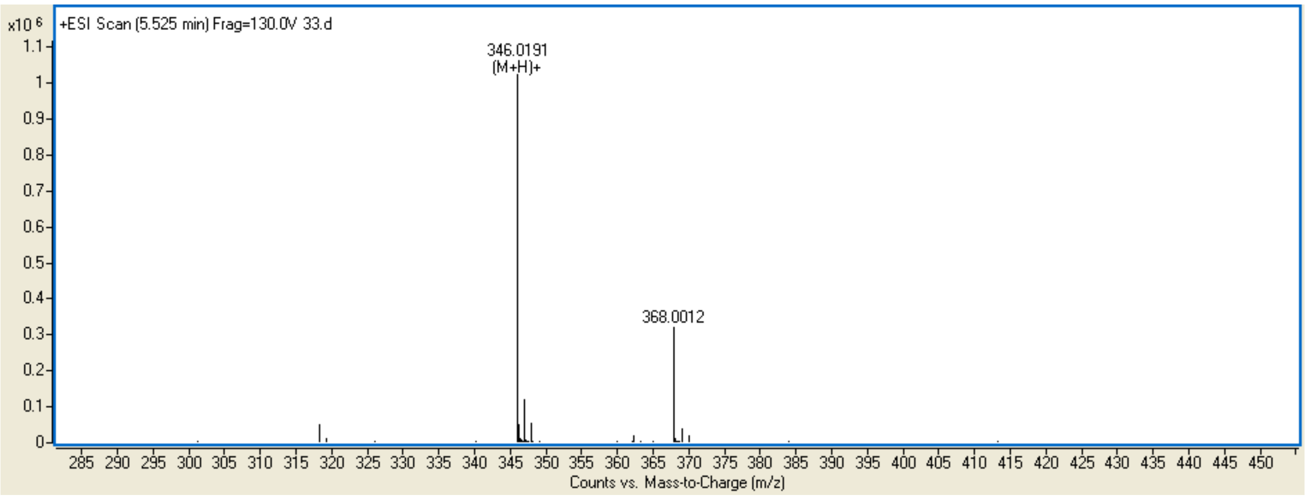

Figure S48. The HR-ESI-MS of compound **7bg**.

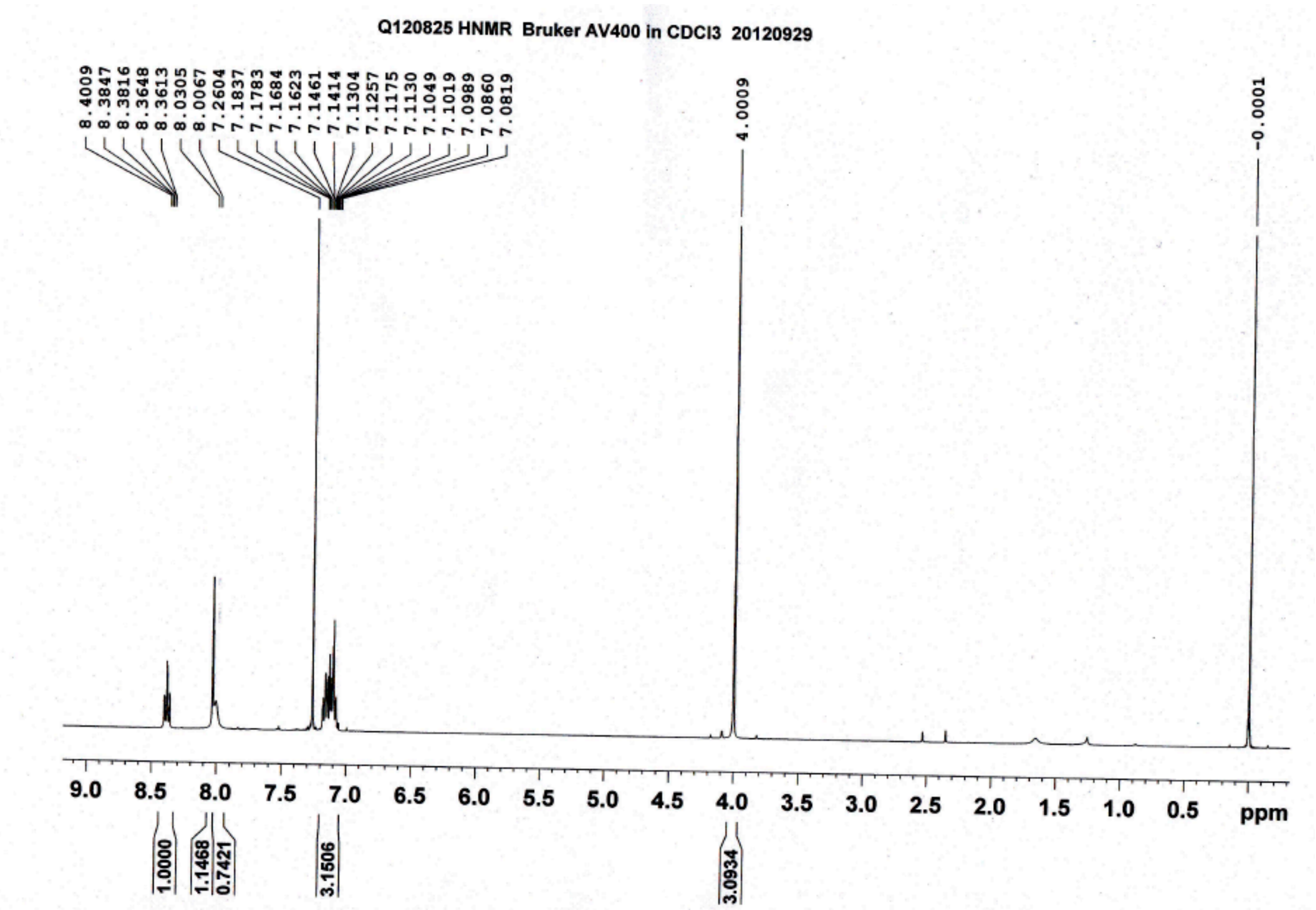

Figure S49. The ^{1}H-NMR ($CDCl_3$, 500 MHz) spectra of compound **7bh**.

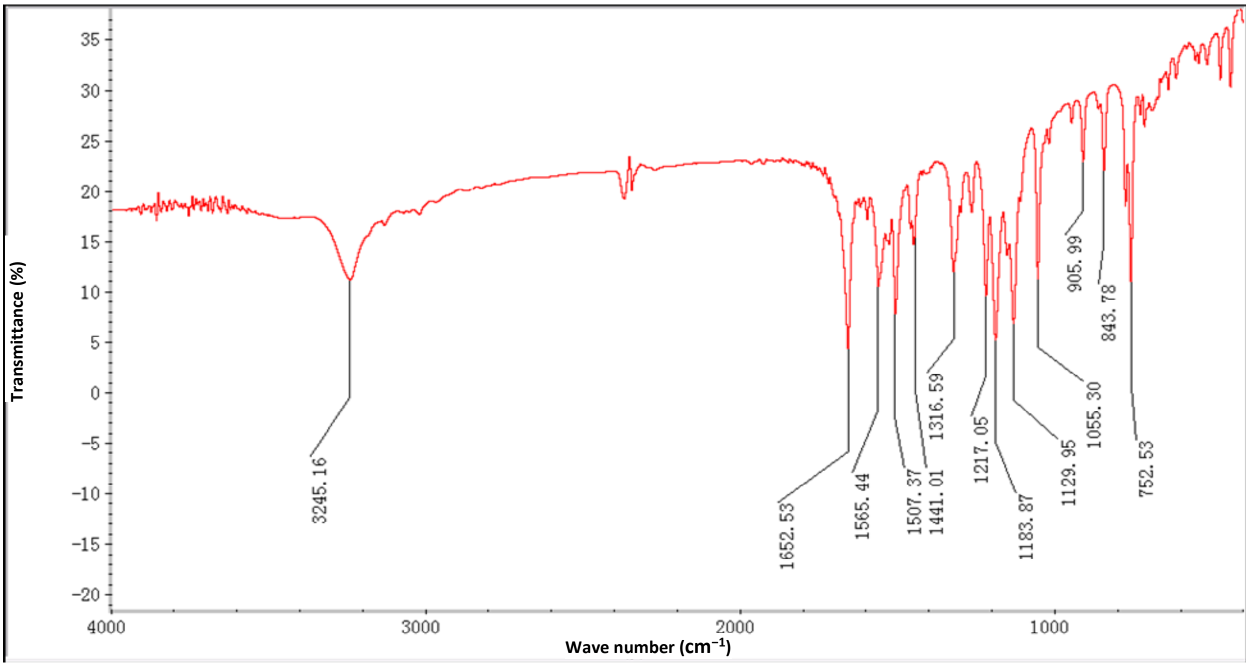

Figure S50. The IR spectra of compound **7bh**.

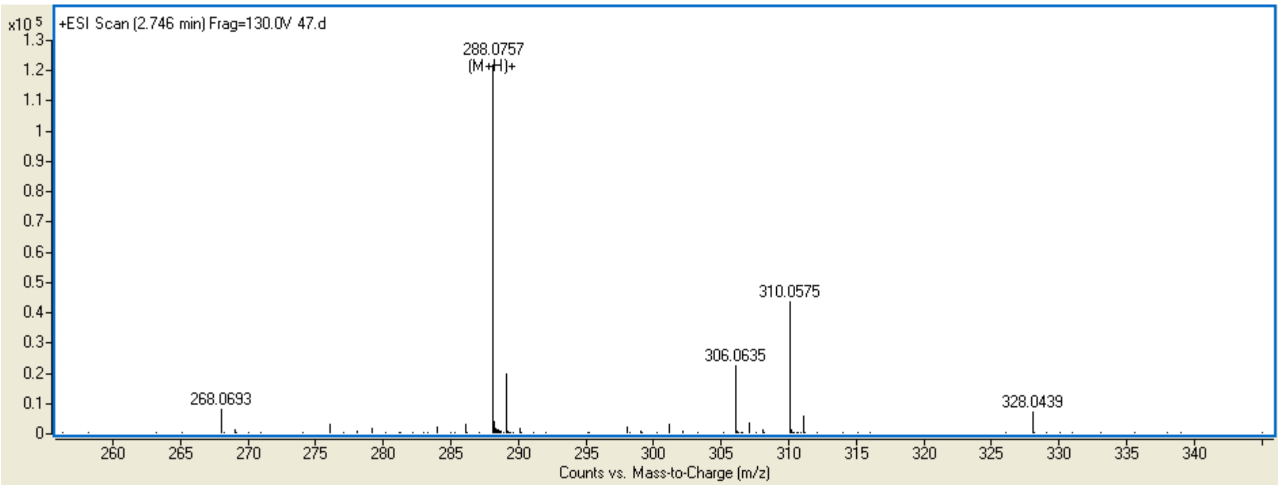

Figure S51. The HR-ESI-MS of compound **7bh**.

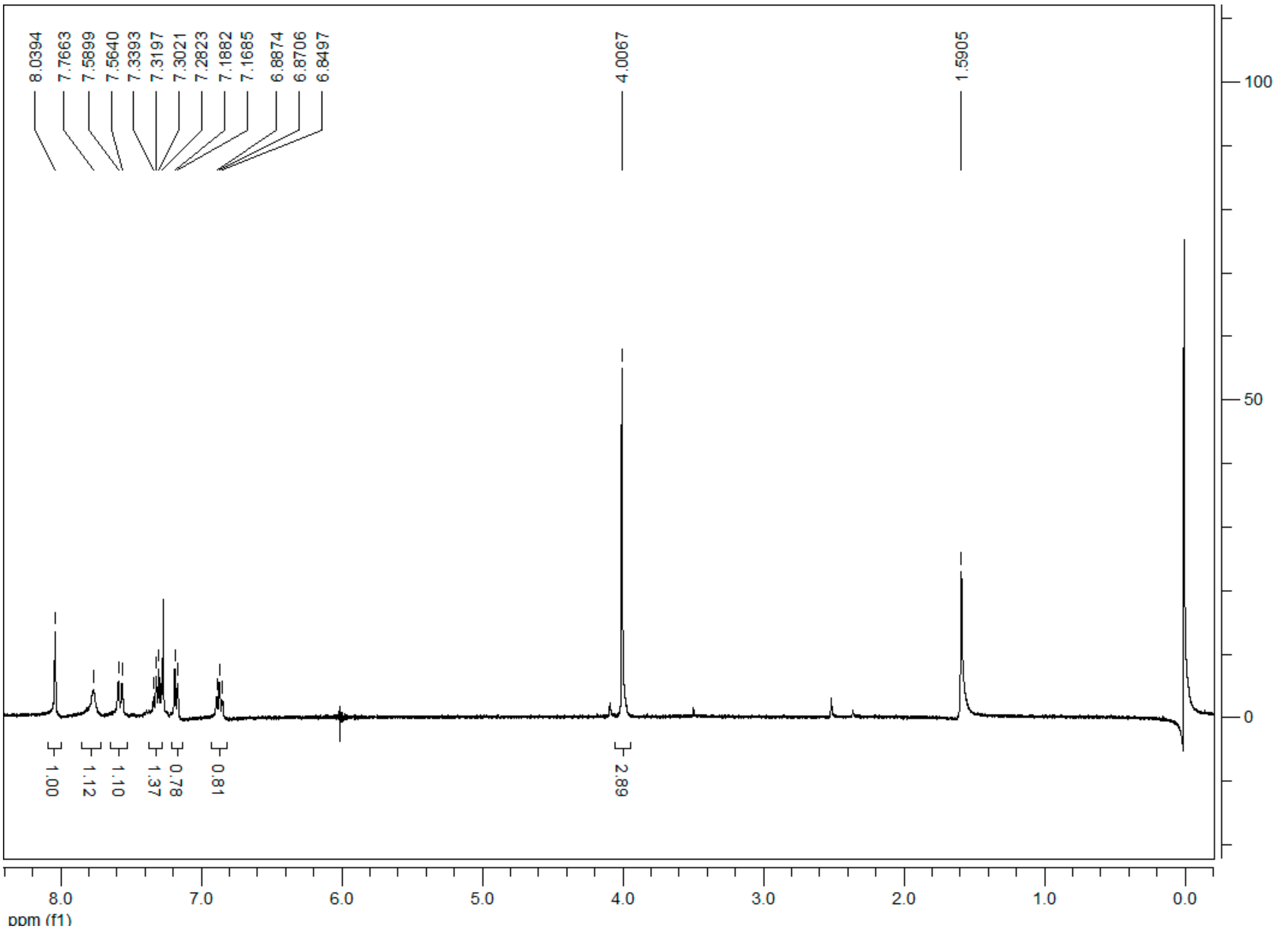

Figure S52. The ^{1}H-NMR ($CDCl_3$, 500 MHz) spectra of compound **7bi**.

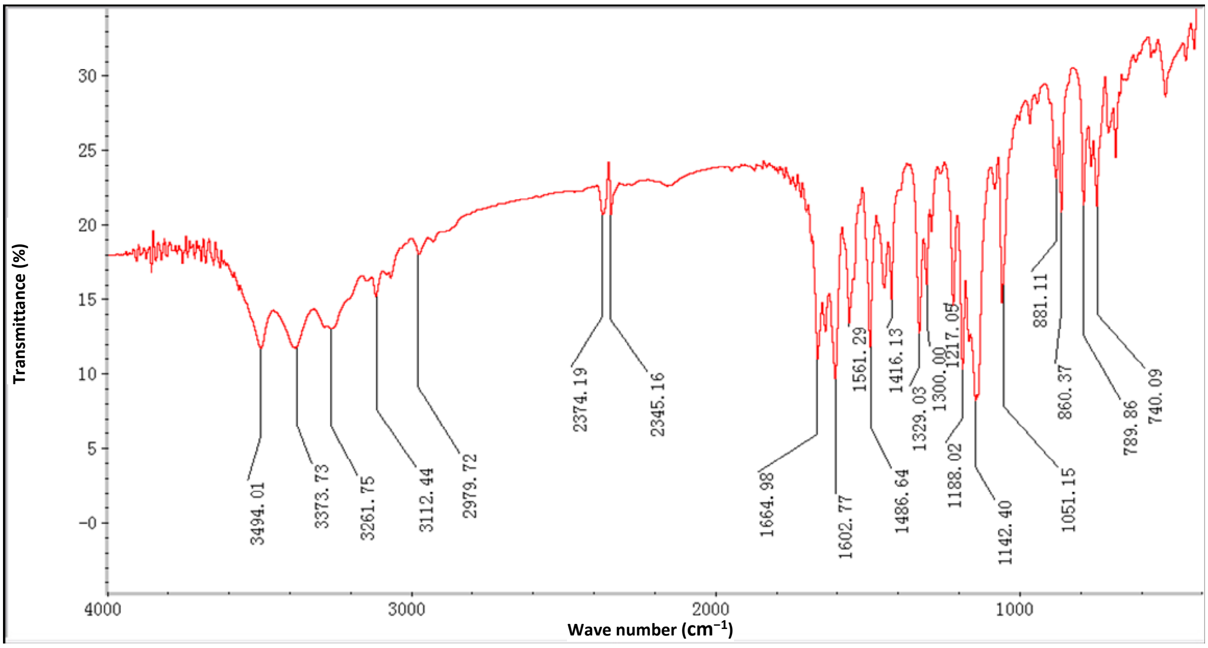

Figure S53. The IR spectra of compound **7bi**.

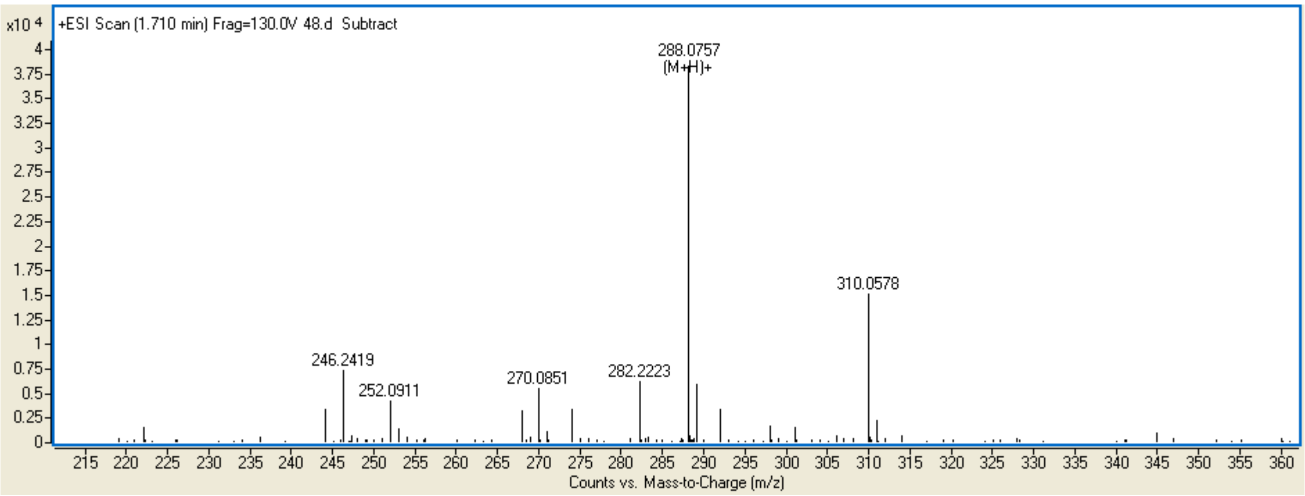

Figure S54. The HR-ESI-MS of compound **7bi**.

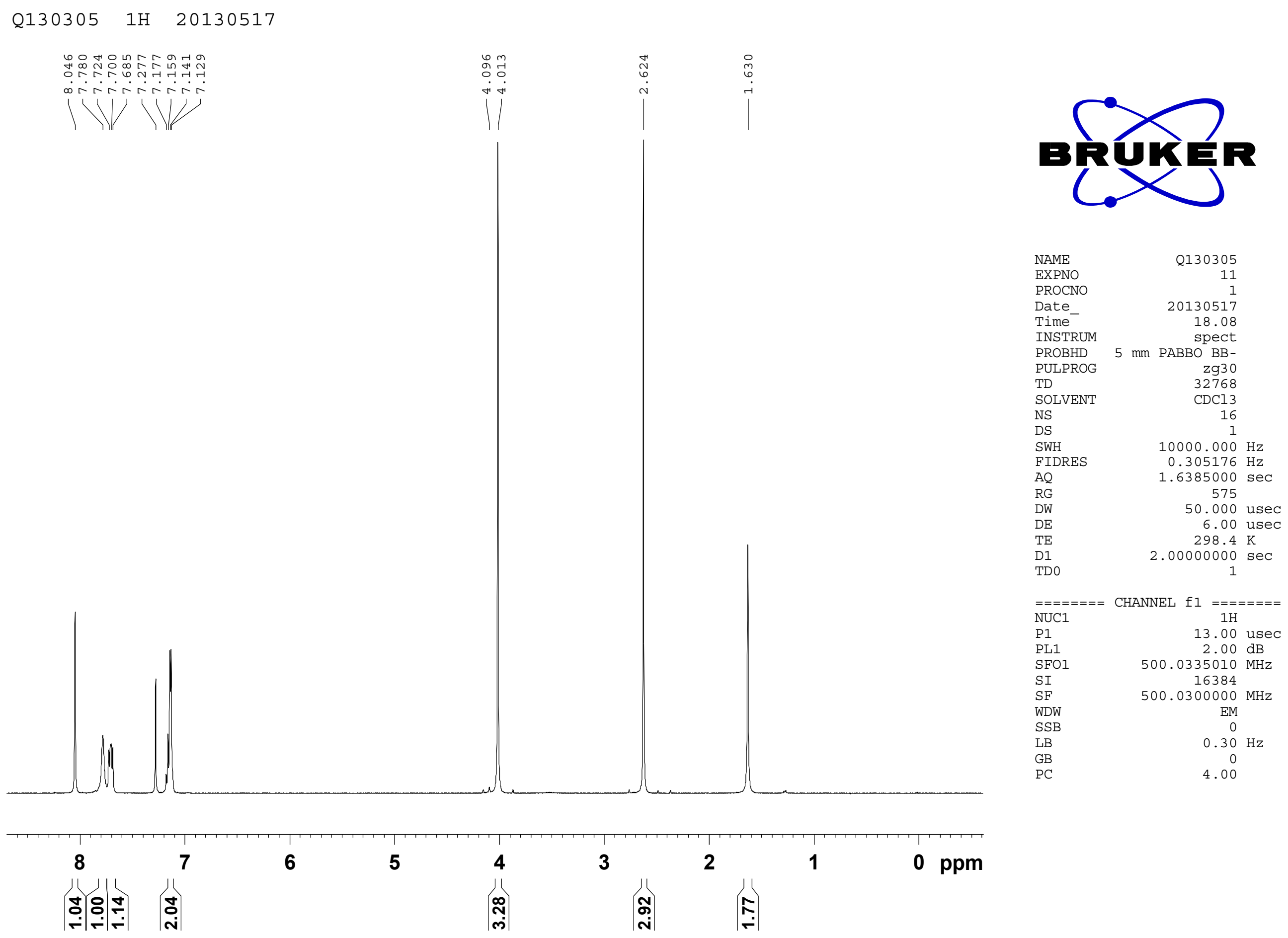

Figure S55. The ^{1}H-NMR ($CDCl_3$, 400 MHz) spectra of compound **7bj**.

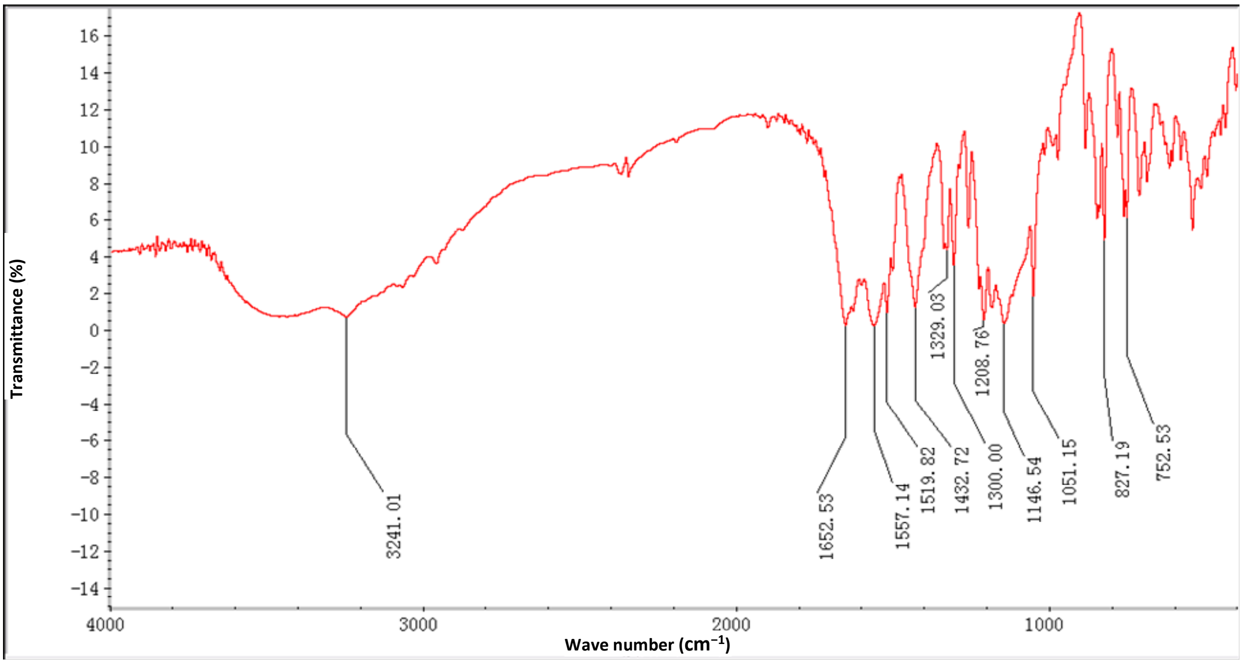

Figure S56. The IR spectra of compound **7bj**.

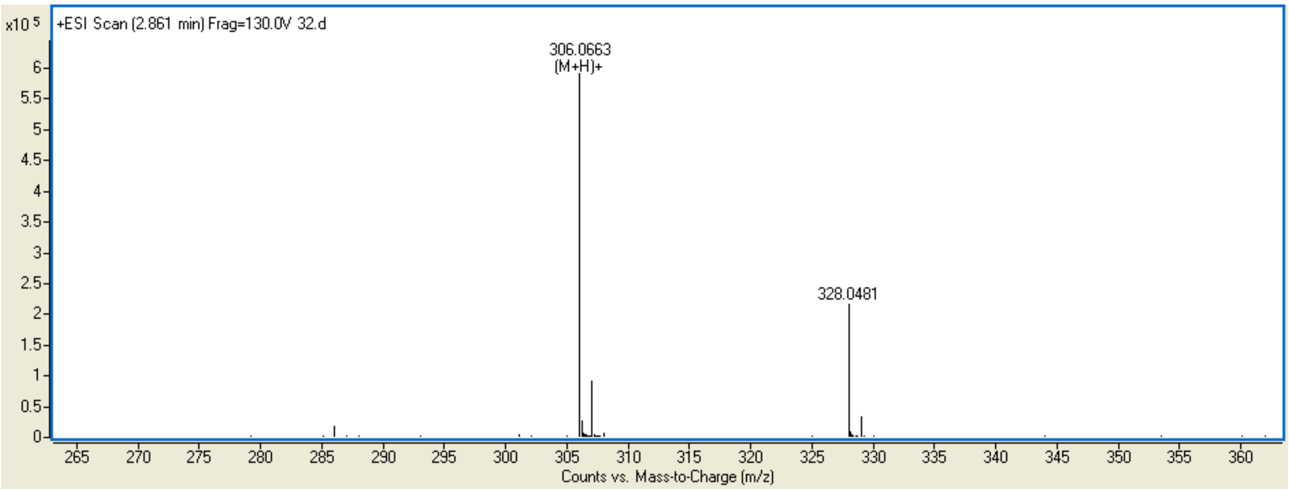

Figure S57. The HR-ESI-MS of compound **7bj**.

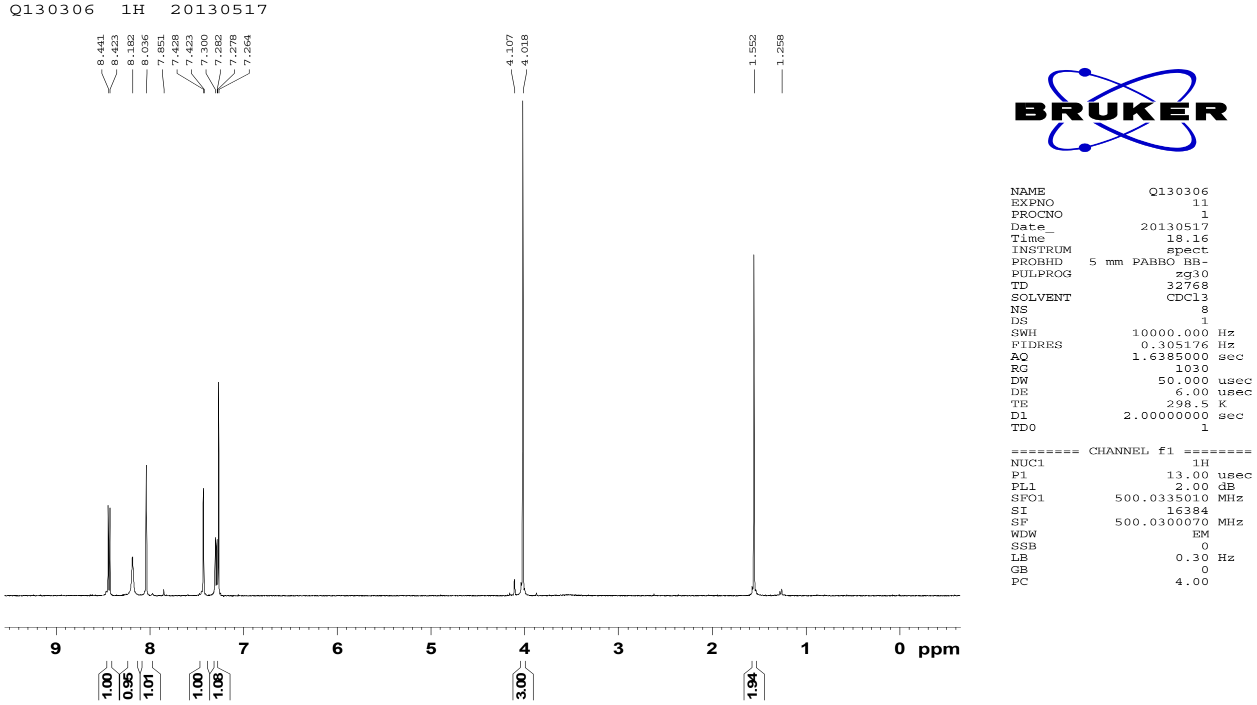

Figure S58. The ^{1}H-NMR ($CDCl_3$, 400 MHz) spectra of compound **7bk**.

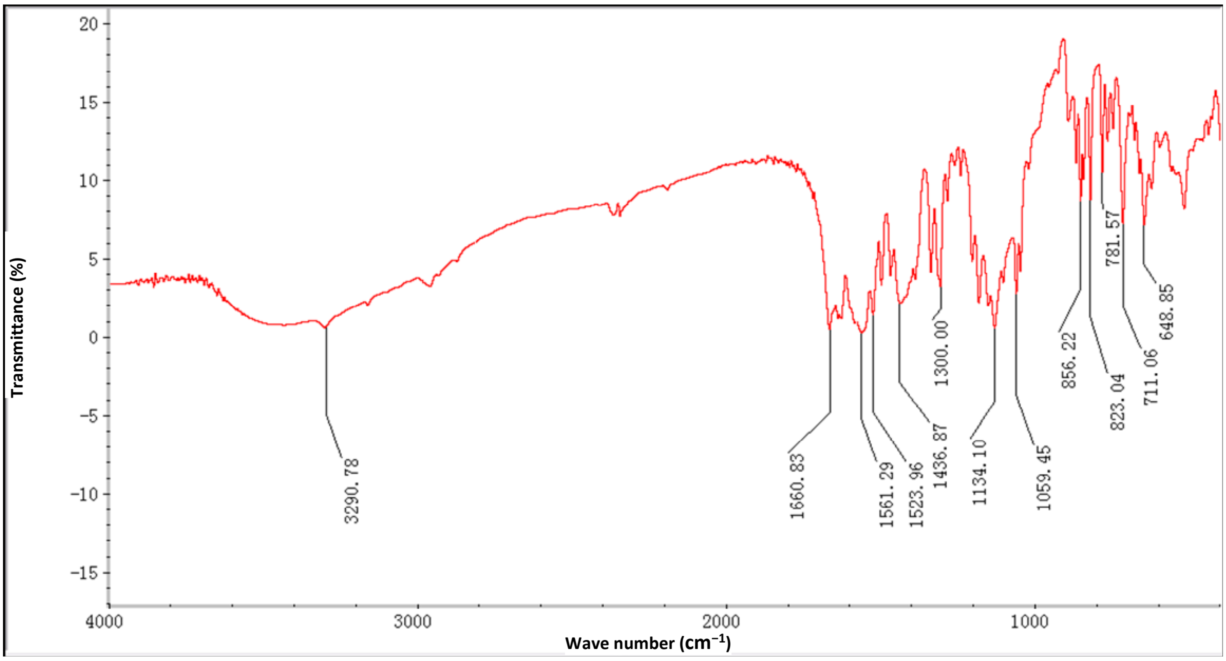

Figure S59. The IR spectra of compound **7bk**.

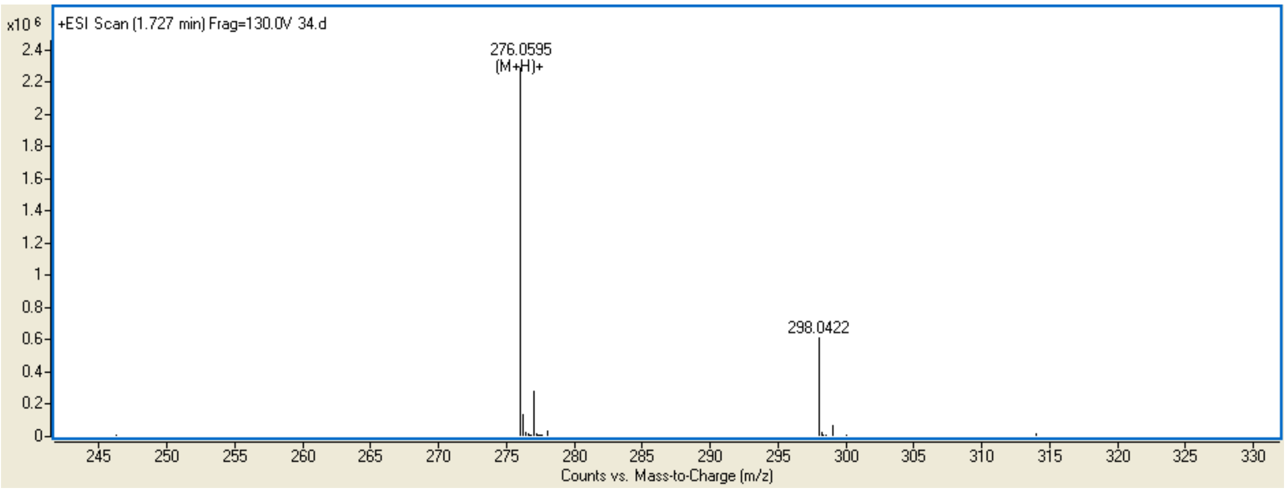

Figure S60. The HR-ESI-MS of compound **7bk**.
